# Retrospective observational study comparing the international hip dysplasia institute classification with the Tonnis classification of developmental dysplasia of the hip

**DOI:** 10.1097/MD.0000000000005902

**Published:** 2017-01-20

**Authors:** Mingyuan Miao, Haiqing Cai, Liwei Hu, Zhigang Wang

**Affiliations:** a Department of Orthopedic Surgery; b Department of Radiology, Shanghai Children's Medical Center, Shanghai Jiao Tong University School of Medicine, Shanghai, China.

**Keywords:** DDH, IHDI classification, Tonnis classification, treatment selection

## Abstract

The Tonnis radiographic classification of developmental dysplasia of the hip (DDH) has been widely used. The International Hip Dysplasia Institute (IHDI) classification, a new classification system recently developed by the IHDI, is beginning to be applied to evaluate DDH with the absence of an ossification center. This study aimed to validate its reliability in evaluating DDH with an ossification center and compared the 2 classifications in evaluating all DDH hips. In addition, the prediction values of the 2 classifications on clinical management selection were compared.

In total, the pelvic radiographs of 212 DDH patients (318 hips) between the ages of 6 and 48 months admitted to Shanghai Children's Medical Center between 2007 and 2014 were assessed by 3 observers retrospectively using the 2 classifications. Intraobserver and interobserver agreements were evaluated using the kappa method. We also assessed the correlation of the 2 radiographic classifications in terms of treatment selection.

In total, 216 hips received closed reduction, 61 hips received open reduction, and 41 hips received pelvic osteotomy. Both classifications showed excellent intraobserver and interobserver reliability. However, the IHDI demonstrated more interobserver reliability, especially for evaluating DDH without an ossification center. Both classifications were found to be relevant in detecting the DDH treatment type (*P* < 0.01). The Tonnis classification was also relevant, especially for evaluating DDH with an ossification center.

The IHDI classification exhibited good practicability in classifying the radiographic severity of DDH compared to the Tonnis classification, particularly in hips without an ossification center. Like the Tonnis classification, the IHDI classification can predict treatment plans. Therefore, the IHDI classification seems to be the upgraded version of the Tonnis classification.

## Abstract 2

Supplemental Digital Content is available in the text

## 1 Introduction

Developmental dysplasia of the hip (DDH) is a very common disorder in the pediatric population, with an incidence of approximately 3 or 4 per 1000 live births.^[1]^ Physical examinations are important for diagnosing DDH in its early stages; however, not all cases of DDH are detectable by clinical examination. Imaging examinations include ultrasonography and radiography, both of which are popular for screening or confirming the diagnosis and the severity classification of DDH.^[2]^ Quantifying the severity of displacement in DDH is important for its diagnosis and treatment. Anteroposterior (AP) pelvic radiographs have replaced the less accurate ultrasonography for screening and imaging in older infants.^[3]^ However, AP pelvic radiographic assessments may be suboptimal or misleading when the ossification of the femoral head of the hips is absent, delayed, or eccentric, as in DDH.

In 1978, Tonnis described a pelvic radiographic classification of DDH depending on the ossification of the femoral head of the hips.^[4]^ Unlike the center-edge angle of Wiberg and the acetabular index angle of Hilgenreiner, the Tonnis classification covers the full spectrum of DDH severity using plain radiographs, as the Graf subtypes are well established for ultrasounds.^[4–7]^ The Tonnis classification has previously been shown to be predictive of treatment success and the need for secondary surgery.^[8,9]^ However, this method relies on the relative position of the ossific nucleus to Perkin's line (P-line) and Hilgenreiner's line (H-line); therefore, it has limitations in that it relies on the presence of an ossific nucleus, which may not be apparent or may be eccentric, and about whose centricity assumptions must be made. Therefore, this limitation can make the application of the Tonnis classification without the presence of an ossification center quite difficult and potentially unreliable.

Recently, the International Hip Dysplasia Institute (IHDI) proposed an alternative classification system with a wider application than the Tonnis classification. The IHDI classification uses the midpoint of proximal femoral metaphysis as a landmark reference and can therefore be applied to all ages of the pediatric population.^[10]^

The purposes of this investigation were to validate the IHDI classification and compare the reliability of the Tonnis classification with the IHDI classification for evaluating DDH patients, including all the proximal femoral ossification centers, at a single institution with 3 observers and to compare the prognostic ability of the Tonnis classification with the IHDI classification for DDH patients using the clinical outcomes of a retrospective database.

## 2 Methods

Our study was approved by the ethics committee of the Shanghai Children's Medical Center at Shanghai Jiao Tong University. All the children's legal guardians gave written and informed consent.

All the pediatric patients who presented with DDH at Shanghai Children's Medical Center between January 2007 and December 2014 were retrospectively reviewed. The obtained medical data of the pediatric patients included their sex, age, the side of the suffering hip, type of treatment, and AP pelvic radiographs. All the hip x-ray examinations were taken within 2 weeks after beginning DDH treatment.

We identified 239 patients with diagnoses of idiopathic DDH who received treatment between the ages of 6 and 48 months. All hips were included to analyze the reliability of the Tonnis classification compared to that of the IHDI classification. In total, 318 hips were treated by closed reduction, open reduction, or pelvic osteotomy, including combined pelvic and femoral osteotomy.^[11,12]^ All patients had a minimum of an 18-month follow-up and complete clinical records. The correlation of the 2 radiographic classifications in terms of treatment selection was also assessed.

The Tonnis classification and the IHDI classification as reported by Tonnis and Narayanan, respectively, were used.^[4,10]^ The Tonnis classification was assessed according to the relative position of the femoral proximal ossific nucleus to Perkin's line (P-line) and the superolateral margin of the acetabulum line (SMA-line). The P-line is a perpendicular line from the superolateral margin of the acetabulum and the SMA-line is a single line drawn through the superolateral margin of the acetabulum bilaterally. The Tonnis classification was utilized according to these definitions as follows. Grade I: the capital femoral ossification center is medial to the P-line. Grade II: the ossification center is lateral to the P-line but below the SMA-line. Grade III: the ossification center is near or level with the SMA-line. Grade IV: the ossification center is above the SMA-line.^[4]^ This measurement relies on the appearance of an ossification center, which is often eccentric or delayed in DDH hips. When the capital femoral ossification center was absent, the observers assumed the location of the ossific nucleus.^[13]^

The IHDI classification uses the H-point as a landmark reference to determine the location of the hip, which is defined as the midpoint of the superior margin of proximal femoral metaphysis that replaces the ossific nucleus. As in the Tonnis classification, the H-line is drawn bilaterally through the top of the triradiate cartilage in the IHDI classification. The standard P-line is then drawn perpendicular at the superolateral margin of the acetabulum. However, unlike in the Tonnis classification, an additional diagonal line (D-line) is then drawn 45 degrees from the junction of Hilgenreiner's line (H-line) and the P-line. The H-line is a single line drawn through the top of the triradiate radiate cartilage bilaterally. The relation of the H-point to these 3 lines determines the IHDI grade. In an IHDI grade I hip, the H-point is at or medial to the P-line. In an IHDI grade II hip, the H-point is lateral to the P-line and at or medial to the D-line. In an IHDI grade III hip, the H-point is lateral to the D-line and at or inferior to the H-line. Finally, in an IHDI grade IV hip, the H-point is superior to the H-line.

The 3 observers were asked to classify each preoperative radiograph independently with both the Tonnis classification and the IHDI classification and repeated this classification 2 weeks later. The 3 observers included 1 resident pediatric orthopedic surgeon, 1 radiologist, and 1 experienced hip research professor. They were blind to one another's assessments and to the identities of the patients.

### 2.1 Statistical analyses

All statistical analyses were performed using the SAS software (version 9.3, SAS Institute, Cary, NC). The statistical analyses included the use of kappa values with a 95% confidence interval to compare the reliability of the Tonnis classification with that of the IHDI classification.^[14]^ We also evaluated their correlation in different ossific nucleus conditions and treatments using kappa values. The chi-square test was used to obtain frequency data on the treatment type with both the Tonnis and IHDI classifications. The significance levels were set at *P* < 0.05, *P* < 0.01, and *P* < 0.001.

## 3 Results

In total, 318 hips (212 DDH patients) were available for the classification measurement, all of which were classifiable by the 3 observers using the Tonnis and IHDI classifications (see Excel Table, Supplemental Content which illustrates the clinical data of 212 patients). In total, 27 patients with inadequate or unavailable radiographs or misdiagnoses in the course of clinical treatment were excluded. The weighted kappa values of all 318 hips using both the Tonnis and IHDI classifications are presented in Tables 1 and 2, respectively.

**Table 1:**
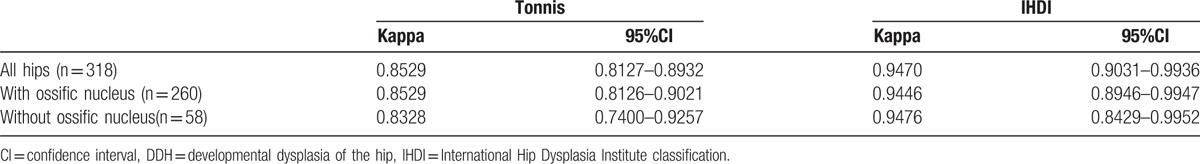
Interobserver agreements for Tonnis and IHDI classification in DDH hips.

**Table 2:**
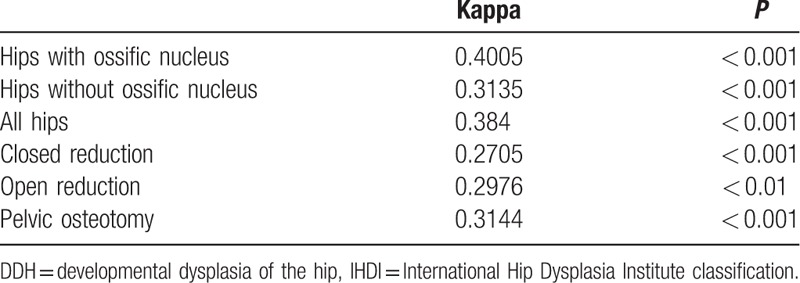
The correlation of Tonnis and IHDI classification in different ossific nucleus and treatment conditions.

The weighted kappa values demonstrated excellent intraobserver agreement for both classifications; the average kappa values of the 3 observers for the Tonnis and IHDI classifications were 0.9652 and 0.9624, respectively. The weighted kappa values also demonstrated good interobserver agreement for both the classifications, but the IHDI showed better agreement (0.9470 compared to 0.8529), especially in hips without an ossific nucleus (0.9476 compared to 0.8328).

In addition, the weighted kappa value of the correlation of the Tonnis classification with the IHDI classification was 0.3840 (*P* < 0.01). The weighted kappa values of the 3 types of treatment (i.e., closed reduction, open reduction, and pelvic osteotomy) were 0.2705, 0.2976, and 0.3144, respectively (*P* < 0.01). We also sought to assess whether one classification was better than the other regarding the subgroups of patients who underwent different types of operations. The chi-square values of the relation of the 2 classifications with the treatment selection were 127.7552 and 102.3886, respectively (*P* < 0.01), which means that both classifications were relevant in detecting the DDH treatment type.

## 4 Discussion

The original intention of the IHDI classification was to remedy the limitations of the Tonnis classification in cases with the disappearance or the eccentric location of the ossific nucleus, as the Tonnis classification depends on the relation of the ossific nucleus to the P-line and the H-line.^[10]^ The IHDI and Tonnis classifications are both practical in the radiographic evaluation of DDH; however, the former classification shows better stability, particularly in evaluating DDH with the disappearance or eccentric location of the ossific nucleus. The IHDI classification can be applied for evaluating DDH regardless of the appearance or disappearance of the ossific nucleus. Therefore, the IHDI classification seems to be the upgraded version of the Tonnis classification.^[15]^

**Figure 1:**
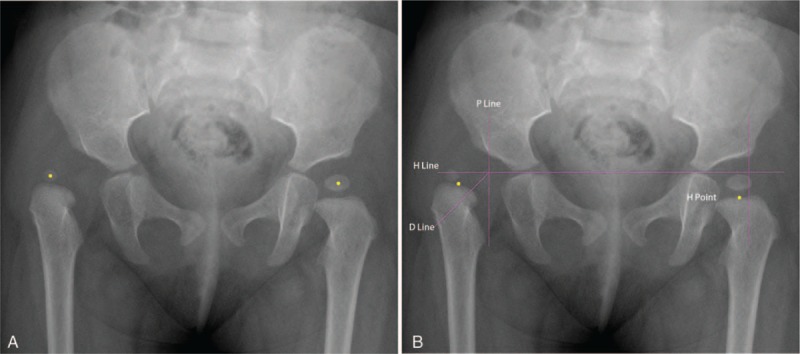
Anteroposterior pelvic radiograph of an infant with right DDH. (A) The right hip was classified as a Tonnis grade III hip and the left hip was classified as a Tonnis grade I hip. The yellow point represented the center in ossific nucleus of femoral head. The right yellow point is located in the lower part of external upper rim of acetabulum but near with the SMA-line, whereas the left yellow point is medial to the P-line. (B) The right hip was classified as an IHDI grade III and the left hip was classified as an IHDI grade I. The yellow point represents the H-point which is defined as the midpoint of the superior margin of proximal femoral metaphysis. The horizontal/vertical/oblique purple lines respectively represent the H-line/P-line/D-line. DDH = developmental dysplasia of the hip, D-line = diagonal line, H-line = Hilgenreiner's line, IHDI = International Hip Dysplasia Institute, P-line = Perkin's line, SMA-line = superolateral margin of the acetabulum lines.

**Figure 2:**
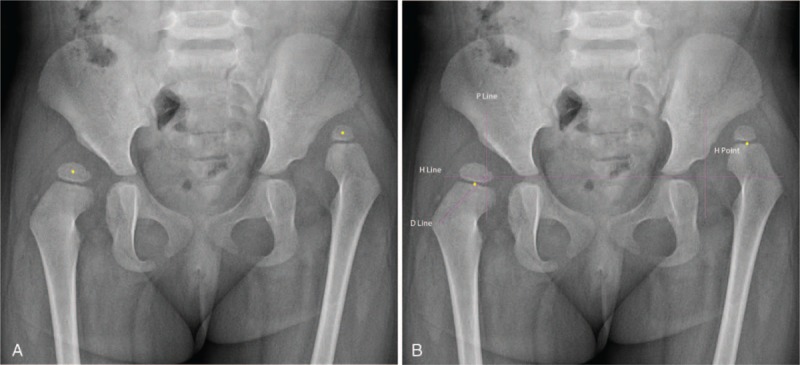
Anteroposterior pelvic radiograph of an infant with bilateral DDH. (A) The right hip was classified as a Tonnis grade II hip and the left hip was classified as a Tonnis grade IV hip. The yellow point represents the center in ossific nucleus of femoral head. The right yellow point is lateral to the P-line but below the SMA-line, whereas the left yellow point is above the SMA-line. (B) The right hip was classified as an IHDI grade III and the left hip was classified as an IHDI grade IV. The yellow point represents the H-point. The horizontal/vertical/oblique purple lines respectively represent the H-line/P-line/D-line. DDH = developmental dysplasia of the hip, D-line = diagonal line, H-line = Hilgenreiner's line, IHDI = International Hip Dysplasia Institute, P-line = Perkin's line, SMA-line = superolateral margin of the acetabulum lines.

There are 3 possible reasons for the better stability of the IHDI classification of DDH compared to the Tonnis classification (Figs. 1 and 2, respectively). First, the IHDI classification judges the superior margin of proximal femoral metaphysis as a line, but the Tonnis classification judges the proximal femoral ossific nucleus as a circle or quasi-circle. The latter encounters difficulty when an edge is vague or irregular, especially in imagined edge situations. Second, the H-point is the midpoint in a metaphysis margin line, but the Tonnis classification determines a circle's center. It is much easier to judge a line and midpoint than a circle's boundary and center, especially in DDH without an ossific nucleus, which implies an imagined circle. Third, the IHDI classification has more accuracy in evaluating IHDI grade II and III hips because the lower outer quadrant is divided into 2 precisely equal parts by the D-line.

Both the IHDI and Tonnis classifications were associated with the treatment type, and the significance level of both was *P* < 0.001, which means that the 2 classifications can predict the operation type.^[16]^ When considering the correlation between the IHDI classification and the Tonnis classification, we found that the kappa value appears better in evaluating DDH with an ossific nucleus in open reduction and in pelvic osteotomy. It is possible that the proximal femoral ossific nucleus becomes clearer and easier to judge with increasing age; however, the H-point is always prone to identification.^[17,18]^

Owing to the different locations of the proximal femoral ossific nucleus and the H-point, there is some disparity between the 2 classifications. Both classifications used the same P-line to distinguish IHDI grade I and grade II hips. Since the H-point is usually on the outside of the proximal femoral ossific nucleus, the IHDI grade II hip is easier reach than the Tonnis grade II hip. The IHDI classification showed an advantage in accuracy in differentiating the classification of grade II and grade III hips because the D-line quantified the lower outer quadrant. The IHDI and Tonnis classifications use the H-line and the SMA-line, respectively, and the distance between the H-line and the SMA-line is usually greater than the longitudinal distance between the H-point and the proximal femoral ossific nucleus; thus, IHDI grade IV hips are easier to reach than Tonnis grade IV hips (Figs. 1 and 2, respectively).

This study has several limitations. First, it is a retrospective observational study. Second, the data of this study were from the single medical center. Third, we have not classified the pelvic radiographs at the endpoint of the follow-up which received the treatment. Comparing to the Tonnis classification, the IHDI may also have some limitation such as requiring a more stringent radiographic position. The H-point is located on the distal side of femoral proximal ossific nucleus, but the femoral proximal ossific nucleus near or located in the center of femoral head, which is similar to the relationship between a circle's center and boundary. When the children thigh is in abduction or adduction position during examination, the displacement of H-point will be significantly greater than ossific nucleus which will leading to obvious misjudgment by the IHDI classification.

In conclusion, the IHDI classification is subjectively easier to use, more accurate, and has favorable interrelater agreement for classifying DDH radiography with the ossific nucleus. The main reason is that the H-point and the D-line make it much easier to determine the severity of DDH accurately. The authors feel that orthopedic surgeons and researchers should consider the IHDI classification as a good alternative to the Tonnis classification when considering DDH treatment.

## Supplementary Material

Supplemental Digital Content
