# Supplementary material for: Retrospective observational study comparing the international hip dysplasia institute classification with the Tonnis classification of developmental dysplasia of the hip
Source: Medicine (Baltimore). 2017 Jan 20;96(3):e5902. doi: 10.1097/MD.0000000000005902 (PMC5279095; doi:10.1097/MD.0000000000005902)
Supplement: Supplemental Digital Content [file medi-96-e5902-s001.doc]

Supplemental Digital Content Legends

The excel table illustrates the clinical data of 212 patients including their sex, age, side of the suffering hip, type of treatment, and radiographic classification.

| N0. | Patient | Sex(1=Male,2=Female) | Months | Side(1=L,2=R) | ossification center(1=without ossification center,2=with ) | Tonnis1 | Tonnis2 | Tonnis3 | IHDI1 | IHDI2 | IHDI3 | Treatment(1=closed reducation,2=open reduction,3=pelvic osteotomy) | Tonnis(1=I,2=II,3=III,4=IV) | IHDI(1=I,2=II,3=III,4=IV) | | |
| --- | --- | --- | --- | --- | --- | --- | --- | --- | --- | --- | --- | --- | --- | --- | --- | --- |
| 1 | 1 | 2 | 8 | 1 | 1 | 2 | 2 | 2 | 2 | 2 | 2 | 1 | 2 | 2 |  |  |
| 2 | 1 | 2 | 8 | 2 | 1 | 2 | 2 | 1 | 2 | 2 | 2 | 1 | 2 | 2 |  |  |
| 3 | 2 | 2 | 10 | 1 | 1 | 2 | 1 | 1 | 2 | 2 | 2 | 1 | 1 | 2 |  |  |
| 4 | 2 | 2 | 10 | 2 | 1 | 1 | 2 | 2 | 2 | 2 | 2 | 1 | 2 | 2 |  |  |
| 5 | 3 | 1 | 11 | 1 | 1 | 1 | 1 | 1 | 2 | 2 | 2 | 1 | 1 | 2 |  |  |
| 6 | 4 | 2 | 13 | 2 | 1 | 2 | 2 | 2 | 2 | 2 | 2 | 1 | 2 | 2 |  |  |
| 7 | 5 | 1 | 6 | 1 | 1 | 3 | 3 | 3 | 3 | 3 | 3 | 1 | 3 | 3 |  |  |
| 8 | 5 | 1 | 6 | 2 | 1 | 3 | 3 | 3 | 3 | 3 | 3 | 1 | 3 | 3 |  |  |
| 9 | 6 | 2 | 9 | 1 | 1 | 1 | 1 | 2 | 2 | 2 | 2 | 1 | 1 | 2 |  |  |
| 10 | 7 | 2 | 7 | 1 | 1 | 2 | 2 | 2 | 2 | 2 | 2 | 1 | 2 | 2 |  |  |
| 11 | 7 | 2 | 7 | 2 | 1 | 2 | 2 | 2 | 2 | 2 | 2 | 1 | 2 | 2 |  |  |
| 12 | 8 | 2 | 7 | 2 | 1 | 1 | 1 | 2 | 2 | 2 | 1 | 1 | 1 | 2 |  |  |
| 13 | 9 | 2 | 16 | 1 | 1 | 1 | 1 | 1 | 2 | 2 | 2 | 1 | 1 | 2 |  |  |
| 14 | 9 | 2 | 16 | 2 | 1 | 1 | 1 | 1 | 2 | 2 | 2 | 1 | 1 | 2 |  |  |
| 15 | 10 | 2 | 6 | 1 | 1 | 3 | 3 | 3 | 4 | 4 | 4 | 1 | 3 | 4 |  |  |
| 16 | 10 | 2 | 6 | 2 | 1 | 3 | 3 | 3 | 4 | 4 | 4 | 1 | 3 | 4 |  |  |
| 17 | 11 | 1 | 7 | 1 | 1 | 2 | 2 | 2 | 3 | 3 | 3 | 1 | 2 | 3 |  |  |
| 18 | 11 | 1 | 7 | 2 | 1 | 2 | 2 | 2 | 3 | 3 | 3 | 1 | 2 | 3 |  |  |
| 19 | 12 | 2 | 12 | 1 | 1 | 2 | 2 | 2 | 2 | 2 | 2 | 1 | 2 | 2 |  |  |
| 20 | 13 | 2 | 8 | 1 | 1 | 2 | 2 | 2 | 3 | 3 | 3 | 1 | 2 | 3 |  |  |
| 21 | 13 | 2 | 8 | 2 | 1 | 3 | 3 | 3 | 4 | 4 | 4 | 1 | 3 | 4 |  |  |
| 22 | 14 | 1 | 17 | 1 | 1 | 2 | 2 | 1 | 2 | 2 | 2 | 1 | 2 | 2 |  |  |
| 23 | 14 | 1 | 17 | 2 | 1 | 2 | 1 | 2 | 2 | 2 | 2 | 1 | 2 | 2 |  |  |
| 24 | 15 | 2 | 7 | 2 | 1 | 2 | 2 | 2 | 3 | 3 | 3 | 1 | 2 | 3 |  |  |
| 25 | 16 | 2 | 12 | 1 | 1 | 2 | 2 | 2 | 2 | 2 | 2 | 1 | 2 | 2 |  |  |
| 26 | 17 | 2 | 8 | 1 | 1 | 2 | 2 | 3 | 3 | 3 | 3 | 1 | 2 | 3 |  |  |
| 27 | 17 | 2 | 8 | 2 | 1 | 3 | 3 | 3 | 3 | 3 | 3 | 1 | 3 | 3 |  |  |
| 28 | 18 | 1 | 15 | 1 | 1 | 2 | 2 | 2 | 3 | 3 | 3 | 1 | 2 | 3 |  |  |
| 29 | 19 | 2 | 6 | 1 | 1 | 1 | 1 | 1 | 2 | 2 | 2 | 1 | 1 | 2 |  |  |
| 30 | 19 | 2 | 6 | 2 | 1 | 1 | 1 | 2 | 2 | 2 | 2 | 1 | 1 | 2 |  |  |
| 31 | 20 | 1 | 17 | 1 | 1 | 2 | 2 | 2 | 2 | 2 | 2 | 1 | 2 | 2 |  |  |
| 32 | 20 | 1 | 17 | 2 | 1 | 2 | 2 | 2 | 2 | 2 | 2 | 1 | 2 | 2 |  |  |
| 33 | 21 | 1 | 11 | 2 | 1 | 2 | 2 | 2 | 2 | 2 | 3 | 1 | 2 | 2 |  |  |
| 34 | 22 | 2 | 13 | 1 | 1 | 1 | 1 | 1 | 2 | 2 | 2 | 1 | 1 | 2 |  |  |
| 35 | 22 | 2 | 13 | 2 | 1 | 1 | 1 | 1 | 2 | 2 | 2 | 1 | 1 | 2 |  |  |
| 36 | 23 | 2 | 9 | 1 | 1 | 2 | 2 | 3 | 3 | 3 | 3 | 1 | 2 | 3 |  |  |
| 37 | 23 | 2 | 9 | 2 | 1 | 2 | 2 | 2 | 3 | 2 | 3 | 1 | 2 | 3 |  |  |
| 38 | 24 | 1 | 10 | 2 | 1 | 2 | 2 | 2 | 2 | 3 | 3 | 1 | 2 | 3 |  |  |
| 39 | 25 | 2 | 12 | 1 | 1 | 3 | 3 | 3 | 3 | 3 | 3 | 1 | 3 | 3 |  |  |
| 40 | 25 | 2 | 12 | 2 | 1 | 3 | 3 | 3 | 3 | 3 | 3 | 1 | 3 | 3 |  |  |
| 41 | 26 | 1 | 6 | 1 | 1 | 1 | 1 | 1 | 2 | 2 | 2 | 1 | 1 | 2 |  |  |
| 42 | 26 | 1 | 6 | 2 | 1 | 1 | 1 | 2 | 2 | 2 | 2 | 1 | 1 | 2 |  |  |
| 43 | 27 | 1 | 14 | 1 | 1 | 2 | 2 | 3 | 3 | 3 | 2 | 1 | 2 | 3 |  |  |
| 44 | 28 | 2 | 9 | 2 | 1 | 4 | 4 | 4 | 4 | 4 | 4 | 1 | 4 | 4 |  |  |
| 45 | 29 | 2 | 11 | 1 | 1 | 2 | 2 | 2 | 2 | 2 | 2 | 1 | 2 | 2 |  |  |
| 46 | 29 | 2 | 11 | 2 | 1 | 2 | 2 | 2 | 2 | 2 | 2 | 1 | 2 | 2 |  |  |
| 47 | 30 | 2 | 13 | 1 | 1 | 2 | 2 | 2 | 3 | 3 | 3 | 1 | 2 | 3 |  |  |
| 48 | 30 | 2 | 13 | 2 | 1 | 2 | 2 | 2 | 2 | 3 | 3 | 1 | 2 | 3 |  |  |
| 49 | 31 | 2 | 9 | 1 | 1 | 3 | 3 | 3 | 4 | 4 | 4 | 1 | 3 | 4 |  |  |
| 50 | 31 | 2 | 9 | 2 | 1 | 3 | 3 | 3 | 4 | 4 | 4 | 1 | 3 | 4 |  |  |
| 51 | 32 | 1 | 15 | 2 | 1 | 2 | 2 | 2 | 3 | 3 | 3 | 1 | 2 | 3 |  |  |
| 52 | 33 | 1 | 6 | 1 | 1 | 2 | 2 | 2 | 3 | 3 | 3 | 1 | 2 | 3 |  |  |
| 53 | 33 | 1 | 6 | 2 | 1 | 3 | 2 | 2 | 3 | 3 | 3 | 1 | 2 | 3 |  |  |
| 54 | 34 | 2 | 9 | 1 | 1 | 2 | 2 | 2 | 3 | 3 | 3 | 1 | 2 | 3 |  |  |
| 55 | 34 | 2 | 9 | 2 | 1 | 2 | 2 | 2 | 3 | 3 | 3 | 1 | 2 | 3 |  |  |
| 56 | 35 | 1 | 13 | 1 | 1 | 3 | 3 | 3 | 3 | 3 | 3 | 1 | 3 | 3 |  |  |
| 57 | 36 | 2 | 16 | 1 | 1 | 2 | 2 | 2 | 2 | 2 | 2 | 1 | 2 | 2 |  |  |
| 58 | 37 | 1 | 18 | 1 | 1 | 1 | 1 | 1 | 2 | 2 | 2 | 1 | 1 | 2 |  |  |
| 59 | 37 | 1 | 18 | 2 | 1 | 1 | 1 | 1 | 2 | 2 | 2 | 1 | 1 | 2 |  |  |
| 60 | 38 | 2 | 7 | 1 | 1 | 3 | 3 | 3 | 4 | 4 | 4 | 1 | 3 | 4 |  |  |
| 61 | 39 | 1 | 11 | 1 | 1 | 2 | 2 | 2 | 3 | 3 | 3 | 1 | 2 | 3 |  |  |
| 62 | 39 | 1 | 11 | 2 | 1 | 2 | 2 | 2 | 3 | 3 | 3 | 1 | 2 | 3 |  |  |
| 63 | 40 | 2 | 8 | 2 | 1 | 3 | 3 | 2 | 3 | 3 | 3 | 1 | 3 | 3 |  |  |
| 64 | 41 | 2 | 10 | 1 | 1 | 2 | 2 | 2 | 3 | 3 | 3 | 1 | 2 | 3 |  |  |
| 65 | 42 | 2 | 11 | 1 | 1 | 2 | 2 | 2 | 3 | 3 | 3 | 1 | 2 | 3 |  |  |
| 66 | 42 | 2 | 11 | 2 | 1 | 3 | 3 | 3 | 4 | 4 | 4 | 1 | 3 | 4 |  |  |
| 67 | 43 | 2 | 15 | 1 | 1 | 2 | 2 | 2 | 3 | 3 | 3 | 1 | 2 | 3 |  |  |
| 68 | 43 | 2 | 15 | 2 | 1 | 1 | 1 | 2 | 2 | 2 | 2 | 1 | 1 | 2 |  |  |
| 69 | 44 | 1 | 13 | 1 | 1 | 1 | 1 | 1 | 2 | 2 | 2 | 1 | 1 | 2 |  |  |
| 70 | 45 | 2 | 14 | 1 | 1 | 2 | 2 | 2 | 2 | 2 | 2 | 1 | 2 | 2 |  |  |
| 71 | 45 | 2 | 14 | 2 | 1 | 2 | 2 | 2 | 3 | 3 | 3 | 1 | 2 | 3 |  |  |
| 72 | 46 | 1 | 6 | 1 | 1 | 3 | 3 | 2 | 3 | 3 | 3 | 1 | 3 | 3 |  |  |
| 73 | 46 | 1 | 6 | 2 | 1 | 3 | 3 | 3 | 3 | 3 | 3 | 1 | 3 | 3 |  |  |
| 74 | 47 | 2 | 11 | 1 | 1 | 2 | 2 | 2 | 3 | 3 | 3 | 1 | 2 | 3 |  |  |
| 75 | 47 | 2 | 11 | 2 | 1 | 3 | 3 | 3 | 4 | 4 | 4 | 1 | 3 | 4 |  |  |
| 76 | 48 | 1 | 13 | 2 | 1 | 2 | 2 | 2 | 3 | 3 | 3 | 1 | 2 | 3 |  |  |
| 77 | 49 | 2 | 12 | 2 | 1 | 3 | 3 | 3 | 3 | 3 | 3 | 1 | 3 | 3 |  |  |
| 78 | 50 | 2 | 9 | 1 | 1 | 2 | 2 | 2 | 2 | 2 | 2 | 1 | 2 | 2 |  |  |
| 79 | 51 | 2 | 9 | 2 | 1 | 3 | 3 | 3 | 4 | 3 | 3 | 1 | 3 | 3 |  |  |
| 80 | 52 | 2 | 8 | 2 | 1 | 2 | 2 | 2 | 3 | 3 | 3 | 1 | 2 | 3 |  |  |
| 81 | 53 | 1 | 7 | 1 | 1 | 3 | 3 | 3 | 3 | 3 | 3 | 1 | 3 | 3 |  |  |
| 82 | 53 | 1 | 7 | 2 | 1 | 2 | 2 | 2 | 3 | 3 | 3 | 1 | 2 | 3 |  |  |
| 83 | 54 | 2 | 11 | 1 | 1 | 2 | 2 | 2 | 2 | 2 | 2 | 1 | 2 | 2 |  |  |
| 84 | 55 | 2 | 15 | 1 | 1 | 2 | 2 | 2 | 3 | 3 | 3 | 1 | 2 | 3 |  |  |
| 85 | 56 | 2 | 15 | 2 | 1 | 4 | 4 | 4 | 4 | 4 | 4 | 1 | 4 | 4 |  |  |
| 86 | 57 | 1 | 7 | 1 | 1 | 3 | 3 | 2 | 3 | 3 | 3 | 1 | 3 | 3 |  |  |
| 87 | 58 | 1 | 13 | 2 | 1 | 1 | 1 | 1 | 2 | 2 | 2 | 1 | 1 | 2 |  |  |
| 88 | 59 | 2 | 18 | 1 | 1 | 1 | 1 | 1 | 2 | 2 | 2 | 1 | 1 | 2 |  |  |
| 89 | 59 | 2 | 18 | 2 | 1 | 1 | 1 | 1 | 2 | 2 | 2 | 1 | 1 | 2 |  |  |
| 90 | 60 | 2 | 10 | 1 | 1 | 3 | 3 | 2 | 3 | 3 | 3 | 1 | 3 | 3 |  |  |
| 91 | 60 | 2 | 10 | 2 | 1 | 2 | 2 | 2 | 3 | 3 | 3 | 1 | 2 | 3 |  |  |
| 92 | 61 | 2 | 7 | 1 | 1 | 4 | 4 | 4 | 4 | 4 | 4 | 1 | 4 | 4 |  |  |
| 93 | 61 | 2 | 7 | 2 | 1 | 3 | 3 | 4 | 4 | 4 | 4 | 1 | 3 | 4 |  |  |
| 94 | 62 | 1 | 10 | 2 | 1 | 2 | 2 | 2 | 3 | 3 | 3 | 1 | 2 | 3 |  |  |
| 95 | 63 | 2 | 9 | 1 | 1 | 3 | 3 | 3 | 4 | 4 | 4 | 1 | 3 | 4 |  |  |
| 96 | 64 | 1 | 14 | 1 | 1 | 1 | 1 | 1 | 2 | 2 | 2 | 1 | 1 | 2 |  |  |
| 97 | 65 | 2 | 7 | 1 | 1 | 1 | 2 | 1 | 2 | 2 | 2 | 1 | 1 | 2 |  |  |
| 98 | 65 | 2 | 7 | 2 | 1 | 1 | 1 | 1 | 2 | 2 | 2 | 1 | 1 | 2 |  |  |
| 99 | 66 | 2 | 6 | 2 | 1 | 2 | 2 | 2 | 3 | 3 | 3 | 1 | 2 | 3 |  |  |
| 100 | 67 | 2 | 7 | 1 | 1 | 2 | 2 | 2 | 3 | 3 | 3 | 1 | 2 | 3 |  |  |
| 101 | 67 | 2 | 7 | 2 | 1 | 2 | 2 | 2 | 3 | 3 | 3 | 1 | 2 | 3 |  |  |
| 102 | 68 | 2 | 11 | 2 | 1 | 1 | 2 | 1 | 2 | 2 | 2 | 1 | 1 | 2 |  |  |
| 103 | 69 | 1 | 15 | 1 | 1 | 2 | 2 | 2 | 3 | 3 | 3 | 1 | 2 | 3 |  |  |
| 104 | 70 | 2 | 7 | 1 | 1 | 2 | 2 | 2 | 3 | 3 | 3 | 1 | 2 | 3 |  |  |
| 105 | 70 | 2 | 7 | 2 | 1 | 1 | 1 | 1 | 2 | 2 | 2 | 1 | 1 | 2 |  |  |
| 106 | 71 | 2 | 6 | 1 | 1 | 2 | 2 | 2 | 3 | 3 | 3 | 1 | 2 | 3 |  |  |
| 107 | 71 | 2 | 6 | 2 | 1 | 3 | 3 | 3 | 3 | 3 | 3 | 1 | 3 | 3 |  |  |
| 108 | 72 | 2 | 7 | 1 | 1 | 3 | 3 | 3 | 3 | 3 | 3 | 1 | 3 | 3 |  |  |
| 109 | 72 | 2 | 7 | 2 | 1 | 2 | 2 | 2 | 2 | 2 | 2 | 1 | 2 | 2 |  |  |
| 110 | 73 | 1 | 8 | 2 | 1 | 2 | 2 | 2 | 2 | 2 | 2 | 1 | 2 | 2 |  |  |
| 111 | 74 | 2 | 9 | 1 | 1 | 1 | 1 | 1 | 2 | 2 | 2 | 1 | 1 | 2 |  |  |
| 112 | 75 | 2 | 12 | 1 | 1 | 3 | 2 | 2 | 2 | 2 | 2 | 1 | 2 | 2 |  |  |
| 113 | 75 | 2 | 12 | 2 | 1 | 2 | 2 | 2 | 3 | 3 | 3 | 1 | 2 | 3 |  |  |
| 114 | 76 | 1 | 11 | 2 | 1 | 3 | 3 | 3 | 3 | 3 | 3 | 1 | 3 | 3 |  |  |
| 115 | 77 | 2 | 9 | 1 | 1 | 2 | 2 | 2 | 2 | 2 | 2 | 1 | 2 | 2 |  |  |
| 116 | 77 | 2 | 9 | 2 | 1 | 3 | 3 | 3 | 3 | 3 | 3 | 1 | 3 | 3 |  |  |
| 117 | 78 | 1 | 8 | 1 | 1 | 3 | 3 | 3 | 4 | 4 | 4 | 1 | 3 | 4 |  |  |
| 118 | 78 | 1 | 8 | 2 | 1 | 2 | 2 | 2 | 3 | 3 | 3 | 1 | 2 | 3 |  |  |
| 119 | 79 | 2 | 12 | 1 | 1 | 2 | 2 | 2 | 3 | 2 | 3 | 1 | 2 | 3 |  |  |
| 120 | 79 | 2 | 12 | 2 | 1 | 1 | 1 | 2 | 2 | 2 | 2 | 1 | 1 | 2 |  |  |
| 121 | 80 | 2 | 7 | 2 | 1 | 2 | 2 | 2 | 2 | 2 | 2 | 1 | 2 | 2 |  |  |
| 122 | 81 | 1 | 6 | 1 | 1 | 3 | 3 | 3 | 4 | 4 | 4 | 1 | 3 | 4 |  |  |
| 123 | 81 | 1 | 6 | 2 | 1 | 1 | 1 | 1 | 2 | 2 | 2 | 1 | 1 | 2 |  |  |
| 124 | 82 | 1 | 17 | 2 | 1 | 2 | 2 | 2 | 2 | 2 | 2 | 1 | 2 | 2 |  |  |
| 125 | 83 | 2 | 16 | 1 | 1 | 2 | 2 | 2 | 3 | 3 | 3 | 1 | 2 | 3 |  |  |
| 126 | 84 | 2 | 13 | 1 | 1 | 2 | 2 | 2 | 3 | 3 | 3 | 1 | 2 | 3 |  |  |
| 127 | 84 | 2 | 13 | 2 | 1 | 2 | 2 | 2 | 3 | 3 | 3 | 1 | 2 | 3 |  |  |
| 128 | 85 | 1 | 12 | 1 | 1 | 3 | 3 | 3 | 3 | 3 | 3 | 1 | 3 | 3 |  |  |
| 129 | 86 | 2 | 6 | 1 | 1 | 1 | 1 | 1 | 2 | 2 | 2 | 1 | 1 | 2 |  |  |
| 130 | 86 | 2 | 6 | 2 | 1 | 1 | 1 | 1 | 2 | 2 | 2 | 1 | 1 | 2 |  |  |
| 131 | 87 | 1 | 6 | 1 | 1 | 1 | 2 | 1 | 2 | 2 | 2 | 1 | 1 | 2 |  |  |
| 132 | 87 | 1 | 6 | 2 | 1 | 2 | 2 | 2 | 2 | 3 | 3 | 1 | 2 | 2 |  |  |
| 133 | 88 | 2 | 8 | 2 | 1 | 2 | 2 | 2 | 3 | 3 | 3 | 1 | 2 | 3 |  |  |
| 134 | 89 | 2 | 11 | 1 | 1 | 2 | 2 | 2 | 3 | 3 | 3 | 1 | 2 | 3 |  |  |
| 135 | 89 | 2 | 11 | 2 | 1 | 2 | 2 | 2 | 3 | 3 | 3 | 1 | 2 | 3 |  |  |
| 136 | 90 | 1 | 9 | 1 | 1 | 1 | 1 | 1 | 2 | 2 | 2 | 1 | 1 | 2 |  |  |
| 137 | 90 | 1 | 9 | 2 | 1 | 3 | 3 | 3 | 3 | 3 | 3 | 1 | 3 | 3 |  |  |
| 138 | 91 | 2 | 8 | 1 | 1 | 2 | 2 | 2 | 2 | 3 | 2 | 1 | 2 | 2 |  |  |
| 139 | 92 | 2 | 6 | 1 | 1 | 2 | 2 | 2 | 3 | 3 | 3 | 1 | 2 | 3 |  |  |
| 140 | 92 | 2 | 6 | 2 | 1 | 2 | 2 | 2 | 3 | 3 | 3 | 1 | 2 | 3 |  |  |
| 141 | 93 | 1 | 7 | 2 | 1 | 3 | 3 | 3 | 4 | 4 | 4 | 1 | 3 | 4 |  |  |
| 142 | 94 | 2 | 6 | 1 | 1 | 1 | 1 | 1 | 2 | 2 | 2 | 1 | 1 | 2 |  |  |
| 143 | 94 | 2 | 6 | 2 | 1 | 2 | 2 | 2 | 2 | 2 | 2 | 1 | 2 | 2 |  |  |
| 144 | 95 | 2 | 9 | 2 | 1 | 2 | 2 | 2 | 3 | 3 | 3 | 1 | 2 | 3 |  |  |
| 145 | 96 | 1 | 15 | 1 | 1 | 3 | 3 | 3 | 3 | 3 | 3 | 1 | 3 | 3 |  |  |
| 146 | 97 | 2 | 14 | 1 | 1 | 2 | 2 | 2 | 3 | 3 | 3 | 1 | 2 | 3 |  |  |
| 147 | 97 | 2 | 14 | 2 | 1 | 1 | 1 | 1 | 2 | 2 | 2 | 1 | 1 | 2 |  |  |
| 148 | 98 | 2 | 10 | 1 | 1 | 1 | 1 | 1 | 2 | 2 | 2 | 1 | 1 | 2 |  |  |
| 149 | 98 | 2 | 10 | 2 | 1 | 2 | 2 | 2 | 3 | 3 | 3 | 1 | 2 | 3 |  |  |
| 150 | 99 | 2 | 18 | 1 | 1 | 1 | 1 | 1 | 2 | 2 | 2 | 1 | 1 | 2 |  |  |
| 151 | 99 | 2 | 18 | 2 | 1 | 1 | 1 | 1 | 2 | 2 | 2 | 1 | 1 | 2 |  |  |
| 152 | 100 | 1 | 6 | 1 | 1 | 2 | 2 | 3 | 3 | 3 | 3 | 1 | 2 | 3 |  |  |
| 153 | 100 | 1 | 6 | 2 | 1 | 2 | 2 | 2 | 2 | 2 | 2 | 1 | 2 | 2 |  |  |
| 154 | 101 | 2 | 6 | 1 | 1 | 4 | 4 | 4 | 4 | 4 | 4 | 2 | 4 | 4 |  |  |
| 155 | 101 | 2 | 6 | 2 | 1 | 4 | 4 | 4 | 4 | 4 | 4 | 2 | 4 | 4 |  |  |
| 156 | 102 | 2 | 15 | 2 | 1 | 3 | 3 | 3 | 3 | 3 | 3 | 2 | 3 | 3 |  |  |
| 157 | 103 | 1 | 12 | 1 | 1 | 3 | 3 | 3 | 4 | 4 | 4 | 2 | 3 | 4 |  |  |
| 158 | 104 | 2 | 9 | 1 | 1 | 3 | 3 | 4 | 4 | 4 | 4 | 2 | 3 | 4 |  |  |
| 159 | 105 | 1 | 8 | 1 | 1 | 3 | 3 | 3 | 3 | 3 | 3 | 2 | 3 | 3 |  |  |
| 160 | 105 | 1 | 8 | 2 | 1 | 3 | 3 | 3 | 3 | 3 | 3 | 2 | 3 | 3 |  |  |
| 161 | 106 | 2 | 7 | 1 | 1 | 4 | 4 | 4 | 4 | 4 | 4 | 2 | 4 | 4 |  |  |
| 162 | 107 | 1 | 11 | 1 | 1 | 4 | 4 | 4 | 4 | 4 | 4 | 2 | 4 | 4 |  |  |
| 163 | 107 | 1 | 11 | 2 | 1 | 3 | 3 | 4 | 3 | 3 | 3 | 2 | 3 | 3 |  |  |
| 164 | 108 | 2 | 9 | 1 | 1 | 3 | 3 | 3 | 3 | 3 | 4 | 2 | 3 | 3 |  |  |
| 165 | 108 | 2 | 9 | 2 | 1 | 3 | 3 | 3 | 4 | 4 | 4 | 2 | 3 | 4 |  |  |
| 166 | 109 | 2 | 18 | 2 | 1 | 3 | 3 | 3 | 3 | 3 | 3 | 2 | 3 | 3 |  |  |
| 167 | 110 | 1 | 12 | 1 | 1 | 3 | 3 | 3 | 3 | 3 | 3 | 2 | 3 | 3 |  |  |
| 168 | 110 | 1 | 12 | 2 | 1 | 4 | 4 | 4 | 4 | 4 | 4 | 2 | 4 | 4 |  |  |
| 169 | 111 | 1 | 17 | 2 | 1 | 3 | 3 | 3 | 3 | 3 | 3 | 2 | 3 | 3 |  |  |
| 170 | 112 | 2 | 10 | 1 | 1 | 4 | 4 | 4 | 4 | 4 | 4 | 2 | 4 | 4 |  |  |
| 171 | 112 | 2 | 10 | 2 | 1 | 3 | 3 | 3 | 4 | 4 | 4 | 2 | 3 | 4 |  |  |
| 172 | 113 | 2 | 16 | 1 | 1 | 4 | 4 | 3 | 4 | 4 | 4 | 2 | 4 | 4 |  |  |
| 173 | 114 | 1 | 7 | 1 | 1 | 3 | 3 | 3 | 4 | 4 | 4 | 2 | 3 | 4 |  |  |
| 174 | 114 | 1 | 7 | 2 | 1 | 3 | 3 | 3 | 3 | 3 | 3 | 2 | 3 | 3 |  |  |
| 175 | 115 | 2 | 13 | 2 | 1 | 3 | 3 | 3 | 4 | 4 | 4 | 2 | 3 | 4 |  |  |
| 176 | 116 | 1 | 8 | 1 | 1 | 4 | 4 | 4 | 4 | 4 | 4 | 2 | 4 | 4 |  |  |
| 177 | 116 | 1 | 8 | 2 | 1 | 3 | 3 | 3 | 4 | 4 | 4 | 2 | 3 | 4 |  |  |
| 178 | 117 | 2 | 7 | 1 | 1 | 3 | 3 | 3 | 3 | 3 | 3 | 2 | 3 | 3 |  |  |
| 179 | 118 | 2 | 9 | 1 | 1 | 3 | 3 | 3 | 4 | 4 | 4 | 2 | 3 | 4 |  |  |
| 180 | 118 | 2 | 9 | 2 | 1 | 3 | 3 | 3 | 3 | 3 | 3 | 2 | 3 | 3 |  |  |
| 181 | 119 | 1 | 11 | 1 | 1 | 3 | 4 | 3 | 4 | 4 | 4 | 2 | 3 | 4 |  |  |
| 182 | 119 | 1 | 11 | 2 | 1 | 4 | 4 | 4 | 4 | 4 | 4 | 2 | 4 | 4 |  |  |
| 183 | 120 | 2 | 12 | 2 | 1 | 3 | 3 | 3 | 4 | 4 | 4 | 2 | 3 | 4 |  |  |
| 184 | 121 | 2 | 7 | 1 | 1 | 4 | 4 | 4 | 4 | 4 | 4 | 2 | 4 | 4 |  |  |
| 185 | 122 | 2 | 8 | 1 | 1 | 3 | 3 | 3 | 3 | 3 | 3 | 2 | 3 | 3 |  |  |
| 186 | 123 | 1 | 9 | 2 | 1 | 4 | 4 | 4 | 4 | 4 | 4 | 2 | 4 | 4 |  |  |
| 187 | 124 | 2 | 6 | 1 | 1 | 3 | 3 | 3 | 3 | 3 | 3 | 2 | 3 | 3 |  |  |
| 188 | 124 | 2 | 6 | 2 | 1 | 3 | 3 | 3 | 4 | 4 | 4 | 2 | 3 | 4 |  |  |
| 189 | 125 | 2 | 17 | 1 | 1 | 4 | 4 | 4 | 4 | 4 | 4 | 2 | 4 | 4 |  |  |
| 190 | 126 | 2 | 8 | 2 | 1 | 3 | 3 | 4 | 4 | 4 | 4 | 2 | 3 | 4 |  |  |
| 191 | 127 | 1 | 15 | 1 | 1 | 3 | 3 | 3 | 3 | 3 | 3 | 2 | 3 | 3 |  |  |
| 192 | 127 | 1 | 15 | 2 | 1 | 4 | 4 | 4 | 4 | 4 | 4 | 2 | 4 | 4 |  |  |
| 193 | 128 | 2 | 11 | 1 | 1 | 3 | 3 | 3 | 4 | 4 | 4 | 2 | 3 | 4 |  |  |
| 194 | 129 | 1 | 9 | 1 | 1 | 3 | 3 | 3 | 3 | 3 | 3 | 2 | 3 | 3 |  |  |
| 195 | 129 | 1 | 9 | 2 | 1 | 3 | 3 | 3 | 3 | 4 | 4 | 2 | 3 | 4 |  |  |
| 196 | 130 | 1 | 7 | 2 | 1 | 3 | 3 | 3 | 3 | 3 | 3 | 2 | 3 | 3 |  |  |
| 197 | 131 | 2 | 6 | 1 | 1 | 3 | 3 | 3 | 4 | 4 | 4 | 2 | 3 | 4 |  |  |
| 198 | 131 | 2 | 6 | 2 | 1 | 4 | 4 | 4 | 4 | 4 | 4 | 2 | 4 | 4 |  |  |
| 199 | 132 | 2 | 16 | 1 | 1 | 4 | 4 | 4 | 4 | 4 | 4 | 2 | 4 | 4 |  |  |
| 200 | 132 | 2 | 16 | 2 | 1 | 3 | 3 | 3 | 4 | 4 | 4 | 2 | 3 | 4 |  |  |
| 201 | 133 | 1 | 9 | 2 | 1 | 3 | 3 | 3 | 3 | 3 | 3 | 2 | 3 | 3 |  |  |
| 202 | 134 | 2 | 12 | 1 | 1 | 3 | 3 | 4 | 4 | 4 | 4 | 2 | 3 | 4 |  |  |
| 203 | 134 | 2 | 12 | 2 | 1 | 4 | 4 | 4 | 4 | 4 | 4 | 2 | 4 | 4 |  |  |
| 204 | 135 | 1 | 25 | 1 | 1 | 2 | 2 | 2 | 3 | 3 | 3 | 3 | 2 | 3 |  |  |
| 205 | 136 | 2 | 19 | 1 | 1 | 3 | 3 | 3 | 4 | 4 | 4 | 3 | 3 | 4 |  |  |
| 206 | 136 | 2 | 19 | 2 | 1 | 4 | 4 | 4 | 4 | 4 | 4 | 3 | 4 | 4 |  |  |
| 207 | 137 | 2 | 37 | 2 | 1 | 2 | 2 | 2 | 3 | 3 | 3 | 3 | 2 | 3 |  |  |
| 208 | 138 | 1 | 43 | 1 | 1 | 3 | 3 | 3 | 3 | 3 | 3 | 3 | 3 | 3 |  |  |
| 209 | 139 | 2 | 22 | 1 | 1 | 3 | 3 | 4 | 4 | 4 | 4 | 3 | 3 | 4 |  |  |
| 210 | 140 | 2 | 28 | 1 | 1 | 2 | 2 | 2 | 3 | 3 | 3 | 3 | 2 | 3 |  |  |
| 211 | 140 | 2 | 28 | 2 | 1 | 3 | 3 | 3 | 3 | 3 | 3 | 3 | 3 | 3 |  |  |
| 212 | 141 | 1 | 47 | 2 | 1 | 3 | 3 | 3 | 3 | 4 | 4 | 3 | 3 | 4 |  |  |
| 213 | 142 | 2 | 20 | 2 | 1 | 3 | 3 | 3 | 4 | 4 | 4 | 3 | 3 | 4 |  |  |
| 214 | 143 | 2 | 25 | 1 | 1 | 2 | 2 | 2 | 2 | 2 | 2 | 3 | 2 | 2 |  |  |
| 215 | 143 | 2 | 25 | 2 | 1 | 2 | 2 | 2 | 3 | 3 | 3 | 3 | 2 | 3 |  |  |
| 216 | 144 | 1 | 33 | 1 | 1 | 2 | 2 | 2 | 3 | 3 | 3 | 3 | 2 | 3 |  |  |
| 217 | 145 | 2 | 39 | 2 | 1 | 2 | 2 | 3 | 3 | 3 | 3 | 3 | 2 | 3 |  |  |
| 218 | 146 | 2 | 25 | 1 | 1 | 3 | 3 | 3 | 3 | 3 | 3 | 3 | 3 | 3 |  |  |
| 219 | 147 | 2 | 28 | 1 | 1 | 2 | 2 | 2 | 2 | 2 | 2 | 3 | 2 | 2 |  |  |
| 220 | 147 | 2 | 28 | 2 | 1 | 2 | 2 | 2 | 3 | 3 | 3 | 3 | 2 | 3 |  |  |
| 221 | 148 | 1 | 40 | 1 | 1 | 2 | 2 | 2 | 3 | 3 | 3 | 3 | 2 | 3 |  |  |
| 222 | 149 | 2 | 19 | 1 | 1 | 2 | 2 | 2 | 2 | 2 | 2 | 3 | 2 | 2 |  |  |
| 223 | 149 | 2 | 19 | 2 | 1 | 2 | 2 | 2 | 3 | 3 | 3 | 3 | 2 | 3 |  |  |
| 224 | 150 | 2 | 23 | 2 | 1 | 3 | 3 | 3 | 4 | 4 | 4 | 3 | 3 | 4 |  |  |
| 225 | 151 | 2 | 29 | 1 | 1 | 4 | 4 | 4 | 4 | 4 | 4 | 3 | 4 | 4 |  |  |
| 226 | 152 | 2 | 24 | 2 | 1 | 2 | 2 | 2 | 3 | 3 | 3 | 3 | 2 | 3 |  |  |
| 227 | 153 | 1 | 35 | 1 | 1 | 2 | 2 | 2 | 2 | 2 | 2 | 3 | 2 | 2 |  |  |
| 228 | 153 | 1 | 35 | 2 | 1 | 2 | 2 | 3 | 3 | 3 | 3 | 3 | 2 | 3 |  |  |
| 229 | 154 | 1 | 41 | 1 | 1 | 4 | 4 | 4 | 4 | 4 | 4 | 3 | 4 | 4 |  |  |
| 230 | 155 | 2 | 48 | 1 | 1 | 2 | 2 | 2 | 3 | 3 | 3 | 3 | 2 | 3 |  |  |
| 231 | 156 | 1 | 30 | 2 | 1 | 2 | 2 | 2 | 3 | 3 | 3 | 3 | 2 | 3 |  |  |
| 232 | 157 | 2 | 19 | 1 | 1 | 3 | 3 | 3 | 3 | 3 | 3 | 3 | 3 | 3 |  |  |
| 233 | 157 | 2 | 19 | 2 | 1 | 2 | 2 | 2 | 3 | 3 | 3 | 3 | 2 | 3 |  |  |
| 234 | 158 | 1 | 27 | 1 | 1 | 2 | 2 | 2 | 3 | 3 | 3 | 3 | 2 | 3 |  |  |
| 235 | 159 | 2 | 29 | 2 | 1 | 3 | 3 | 3 | 4 | 4 | 4 | 3 | 3 | 4 |  |  |
| 236 | 160 | 2 | 31 | 1 | 1 | 2 | 2 | 2 | 3 | 3 | 3 | 3 | 2 | 3 |  |  |
| 237 | 160 | 2 | 31 | 2 | 1 | 4 | 4 | 3 | 4 | 4 | 4 | 3 | 4 | 4 |  |  |
| 238 | 161 | 1 | 36 | 2 | 1 | 3 | 3 | 3 | 3 | 3 | 3 | 3 | 3 | 3 |  |  |
| 239 | 162 | 2 | 38 | 1 | 1 | 3 | 3 | 3 | 4 | 4 | 4 | 3 | 3 | 4 |  |  |
| 240 | 163 | 2 | 23 | 1 | 1 | 4 | 4 | 4 | 4 | 4 | 4 | 3 | 4 | 4 |  |  |
| 241 | 163 | 2 | 23 | 2 | 1 | 2 | 2 | 2 | 2 | 2 | 2 | 3 | 2 | 2 |  |  |
| 242 | 164 | 2 | 19 | 2 | 1 | 3 | 3 | 3 | 3 | 3 | 3 | 3 | 3 | 3 |  |  |
| 243 | 165 | 1 | 25 | 2 | 1 | 2 | 2 | 2 | 2 | 3 | 3 | 3 | 2 | 3 |  |  |
| 244 | 166 | 2 | 44 | 1 | 1 | 2 | 2 | 3 | 3 | 3 | 3 | 3 | 2 | 3 |  |  |
| 245 | 167 | 1 | 13 | 1 | 1 | 1 | 1 | 1 | 2 | 2 | 2 | 1 | 1 | 2 |  |  |
| 246 | 167 | 1 | 13 | 2 | 1 | 3 | 3 | 3 | 4 | 4 | 4 | 2 | 3 | 4 |  |  |
| 247 | 168 | 2 | 9 | 1 | 1 | 3 | 3 | 4 | 4 | 4 | 4 | 2 | 3 | 4 |  |  |
| 248 | 168 | 2 | 9 | 2 | 1 | 2 | 2 | 2 | 2 | 2 | 2 | 1 | 2 | 2 |  |  |
| 249 | 169 | 2 | 16 | 1 | 1 | 2 | 2 | 2 | 2 | 2 | 2 | 1 | 2 | 2 |  |  |
| 250 | 169 | 2 | 16 | 2 | 1 | 3 | 3 | 3 | 4 | 4 | 4 | 2 | 3 | 4 |  |  |
| 251 | 170 | 2 | 7 | 1 | 1 | 2 | 2 | 2 | 3 | 3 | 3 | 1 | 2 | 3 |  |  |
| 252 | 170 | 2 | 7 | 2 | 1 | 3 | 3 | 3 | 4 | 4 | 4 | 2 | 3 | 4 |  |  |
| 253 | 171 | 1 | 11 | 1 | 1 | 1 | 1 | 1 | 2 | 2 | 2 | 1 | 1 | 2 |  |  |
| 254 | 171 | 1 | 11 | 2 | 1 | 3 | 3 | 3 | 4 | 4 | 4 | 2 | 3 | 4 |  |  |
| 255 | 172 | 2 | 14 | 1 | 1 | 4 | 4 | 4 | 4 | 4 | 4 | 2 | 4 | 4 |  |  |
| 256 | 172 | 2 | 14 | 2 | 1 | 1 | 2 | 2 | 2 | 2 | 2 | 1 | 2 | 2 |  |  |
| 257 | 173 | 2 | 9 | 1 | 2 | 1 | 1 | 2 | 2 | 2 | 2 | 1 | 1 | 2 |  |  |
| 258 | 173 | 2 | 9 | 2 | 2 | 1 | 1 | 1 | 2 | 2 | 2 | 1 | 1 | 2 |  |  |
| 259 | 174 | 2 | 8 | 1 | 2 | 1 | 2 | 1 | 2 | 2 | 2 | 1 | 1 | 2 |  |  |
| 260 | 174 | 2 | 8 | 2 | 2 | 1 | 1 | 1 | 2 | 2 | 2 | 1 | 1 | 2 |  |  |
| 261 | 175 | 1 | 7 | 2 | 2 | 2 | 2 | 2 | 2 | 2 | 2 | 1 | 2 | 2 |  |  |
| 262 | 176 | 2 | 9 | 1 | 2 | 2 | 2 | 2 | 2 | 2 | 2 | 1 | 2 | 2 |  |  |
| 263 | 176 | 2 | 9 | 2 | 2 | 2 | 2 | 2 | 3 | 3 | 3 | 1 | 2 | 3 |  |  |
| 264 | 177 | 1 | 11 | 1 | 2 | 2 | 2 | 2 | 3 | 3 | 3 | 1 | 2 | 3 |  |  |
| 265 | 178 | 2 | 6 | 1 | 2 | 3 | 3 | 4 | 4 | 4 | 4 | 1 | 3 | 4 |  |  |
| 266 | 179 | 2 | 13 | 2 | 2 | 2 | 2 | 2 | 3 | 3 | 3 | 1 | 2 | 3 |  |  |
| 267 | 180 | 1 | 8 | 1 | 2 | 3 | 4 | 3 | 4 | 4 | 4 | 1 | 3 | 4 |  |  |
| 268 | 180 | 1 | 8 | 2 | 2 | 4 | 4 | 4 | 4 | 4 | 4 | 1 | 4 | 4 |  |  |
| 269 | 181 | 2 | 6 | 1 | 2 | 4 | 4 | 4 | 3 | 3 | 3 | 1 | 4 | 3 |  |  |
| 270 | 181 | 2 | 6 | 2 | 2 | 2 | 2 | 2 | 2 | 3 | 3 | 1 | 2 | 3 |  |  |
| 271 | 182 | 2 | 12 | 1 | 2 | 3 | 3 | 3 | 4 | 4 | 4 | 1 | 3 | 4 |  |  |
| 272 | 182 | 2 | 12 | 2 | 2 | 1 | 1 | 1 | 2 | 2 | 2 | 1 | 1 | 2 |  |  |
| 273 | 183 | 1 | 7 | 2 | 2 | 2 | 2 | 2 | 3 | 3 | 3 | 1 | 2 | 3 |  |  |
| 274 | 184 | 2 | 10 | 1 | 2 | 1 | 1 | 1 | 2 | 2 | 2 | 1 | 1 | 2 |  |  |
| 275 | 185 | 2 | 7 | 1 | 2 | 2 | 2 | 2 | 3 | 3 | 3 | 1 | 2 | 3 |  |  |
| 276 | 185 | 2 | 7 | 2 | 2 | 3 | 3 | 3 | 3 | 3 | 3 | 1 | 3 | 3 |  |  |
| 277 | 186 | 1 | 8 | 2 | 2 | 2 | 2 | 2 | 3 | 3 | 3 | 1 | 2 | 3 |  |  |
| 278 | 187 | 2 | 6 | 1 | 2 | 3 | 2 | 3 | 3 | 3 | 3 | 1 | 3 | 3 |  |  |
| 279 | 188 | 2 | 13 | 1 | 2 | 3 | 3 | 3 | 3 | 3 | 3 | 1 | 3 | 3 |  |  |
| 280 | 188 | 2 | 13 | 2 | 2 | 3 | 3 | 3 | 3 | 3 | 3 | 1 | 3 | 3 |  |  |
| 281 | 189 | 1 | 9 | 2 | 2 | 2 | 2 | 2 | 3 | 3 | 3 | 1 | 2 | 3 |  |  |
| 282 | 190 | 2 | 8 | 1 | 2 | 4 | 4 | 4 | 4 | 4 | 4 | 1 | 4 | 4 |  |  |
| 283 | 190 | 2 | 8 | 2 | 2 | 2 | 2 | 2 | 3 | 3 | 3 | 1 | 2 | 3 |  |  |
| 284 | 191 | 2 | 10 | 1 | 2 | 2 | 2 | 2 | 2 | 2 | 2 | 1 | 2 | 2 |  |  |
| 285 | 192 | 2 | 6 | 1 | 2 | 2 | 2 | 3 | 3 | 3 | 3 | 1 | 2 | 3 |  |  |
| 286 | 192 | 2 | 6 | 2 | 2 | 2 | 2 | 2 | 3 | 3 | 3 | 1 | 2 | 3 |  |  |
| 287 | 193 | 1 | 8 | 2 | 2 | 1 | 1 | 1 | 2 | 2 | 2 | 1 | 1 | 2 |  |  |
| 288 | 194 | 2 | 6 | 1 | 2 | 1 | 1 | 1 | 2 | 2 | 2 | 1 | 1 | 2 |  |  |
| 289 | 194 | 2 | 6 | 2 | 2 | 2 | 2 | 2 | 2 | 3 | 3 | 1 | 2 | 3 |  |  |
| 290 | 195 | 2 | 7 | 1 | 2 | 3 | 3 | 3 | 3 | 3 | 3 | 1 | 3 | 3 |  |  |
| 291 | 195 | 2 | 7 | 2 | 2 | 3 | 3 | 3 | 4 | 4 | 4 | 1 | 3 | 4 |  |  |
| 292 | 196 | 2 | 11 | 1 | 2 | 2 | 2 | 2 | 3 | 3 | 3 | 1 | 2 | 3 |  |  |
| 293 | 196 | 2 | 11 | 2 | 2 | 1 | 1 | 1 | 2 | 2 | 2 | 1 | 1 | 2 |  |  |
| 294 | 197 | 1 | 12 | 1 | 2 | 3 | 3 | 3 | 4 | 4 | 4 | 1 | 3 | 4 |  |  |
| 295 | 198 | 2 | 15 | 2 | 2 | 2 | 2 | 2 | 2 | 2 | 2 | 1 | 2 | 2 |  |  |
| 296 | 199 | 2 | 7 | 2 | 2 | 2 | 2 | 2 | 3 | 3 | 3 | 1 | 2 | 3 |  |  |
| 297 | 200 | 1 | 6 | 1 | 2 | 2 | 2 | 3 | 3 | 3 | 3 | 1 | 2 | 3 |  |  |
| 298 | 201 | 2 | 8 | 1 | 2 | 3 | 3 | 3 | 4 | 4 | 4 | 1 | 3 | 4 |  |  |
| 299 | 201 | 2 | 8 | 2 | 2 | 1 | 1 | 1 | 2 | 2 | 2 | 1 | 1 | 2 |  |  |
| 300 | 202 | 2 | 11 | 1 | 2 | 2 | 2 | 2 | 3 | 3 | 3 | 1 | 2 | 3 |  |  |
| 301 | 202 | 2 | 11 | 2 | 2 | 3 | 3 | 4 | 4 | 4 | 4 | 1 | 3 | 4 |  |  |
| 302 | 203 | 2 | 7 | 1 | 2 | 2 | 2 | 2 | 2 | 2 | 2 | 1 | 2 | 2 |  |  |
| 303 | 203 | 2 | 7 | 2 | 2 | 2 | 2 | 2 | 3 | 3 | 3 | 1 | 2 | 3 |  |  |
| 304 | 204 | 1 | 9 | 2 | 2 | 3 | 3 | 3 | 4 | 4 | 4 | 1 | 3 | 4 |  |  |
| 305 | 205 | 1 | 11 | 1 | 2 | 3 | 3 | 4 | 4 | 4 | 4 | 1 | 3 | 4 |  |  |
| 306 | 206 | 2 | 9 | 1 | 2 | 3 | 3 | 3 | 3 | 3 | 3 | 1 | 3 | 3 |  |  |
| 307 | 206 | 2 | 9 | 2 | 2 | 2 | 2 | 2 | 2 | 2 | 2 | 1 | 2 | 2 |  |  |
| 308 | 207 | 2 | 8 | 1 | 2 | 2 | 2 | 2 | 2 | 2 | 2 | 1 | 2 | 2 |  |  |
| 309 | 208 | 2 | 8 | 1 | 2 | 3 | 3 | 4 | 4 | 4 | 4 | 2 | 3 | 4 |  |  |
| 310 | 208 | 2 | 8 | 2 | 2 | 2 | 2 | 2 | 3 | 3 | 3 | 1 | 2 | 3 |  |  |
| 311 | 209 | 2 | 10 | 1 | 1 | 1 | 1 | 1 | 2 | 2 | 2 | 1 | 1 | 2 |  |  |
| 312 | 209 | 2 | 10 | 2 | 2 | 4 | 4 | 4 | 4 | 4 | 4 | 2 | 4 | 4 |  |  |
| 313 | 210 | 1 | 7 | 1 | 2 | 3 | 3 | 3 | 4 | 4 | 4 | 2 | 3 | 4 |  |  |
| 314 | 210 | 1 | 7 | 2 | 1 | 2 | 2 | 2 | 3 | 3 | 3 | 1 | 2 | 3 |  |  |
| 315 | 211 | 2 | 6 | 1 | 1 | 2 | 2 | 2 | 3 | 3 | 3 | 1 | 2 | 3 |  |  |
| 316 | 211 | 2 | 6 | 2 | 2 | 4 | 4 | 4 | 4 | 4 | 4 | 2 | 4 | 4 |  |  |
| 317 | 212 | 1 | 11 | 1 | 1 | 1 | 1 | 1 | 2 | 2 | 2 | 1 | 1 | 2 |  |  |
| 318 | 212 | 1 | 11 | 2 | 2 | 3 | 3 | 3 | 4 | 4 | 4 | 2 | 3 | 4 |  |  |

| No. | Patient | Side(1=L,2=R) | ossification centre(1=without ossification center,2=with ) | T1 | T1-2W | T2 | T2-2W | T3 | T3-2W | I1 | I1-2W | I2 | I2-2W | I3 | I3-2W |
| --- | --- | --- | --- | --- | --- | --- | --- | --- | --- | --- | --- | --- | --- | --- | --- |
| 1 | 1 | 1 | 1 | 2 | 2 | 2 | 2 | 2 | 2 | 2 | 2 | 2 | 2 | 2 | 2 |
| 2 | 1 | 2 | 1 | 2 | 2 | 2 | 2 | 1 | 2 | 2 | 2 | 2 | 2 | 2 | 2 |
| 3 | 2 | 1 | 1 | 2 | 2 | 1 | 1 | 1 | 1 | 2 | 2 | 2 | 2 | 2 | 2 |
| 4 | 2 | 2 | 1 | 1 | 1 | 2 | 2 | 2 | 2 | 2 | 2 | 2 | 2 | 2 | 2 |
| 5 | 3 | 1 | 1 | 1 | 1 | 1 | 1 | 1 | 1 | 2 | 2 | 2 | 2 | 2 | 2 |
| 6 | 4 | 2 | 1 | 2 | 2 | 2 | 2 | 2 | 2 | 2 | 2 | 2 | 2 | 2 | 2 |
| 7 | 5 | 1 | 1 | 3 | 3 | 3 | 3 | 3 | 3 | 3 | 3 | 3 | 3 | 3 | 3 |
| 8 | 5 | 2 | 1 | 3 | 3 | 3 | 3 | 3 | 3 | 3 | 3 | 3 | 3 | 3 | 3 |
| 9 | 6 | 1 | 1 | 1 | 1 | 1 | 1 | 2 | 1 | 2 | 2 | 2 | 2 | 2 | 2 |
| 10 | 7 | 1 | 1 | 2 | 2 | 2 | 2 | 2 | 2 | 2 | 2 | 2 | 2 | 2 | 2 |
| 11 | 7 | 2 | 1 | 2 | 2 | 2 | 2 | 2 | 2 | 2 | 2 | 2 | 2 | 2 | 3 |
| 12 | 8 | 2 | 1 | 1 | 1 | 1 | 1 | 2 | 2 | 2 | 2 | 2 | 2 | 1 | 1 |
| 13 | 9 | 1 | 1 | 1 | 1 | 1 | 1 | 1 | 1 | 2 | 2 | 2 | 2 | 2 | 2 |
| 14 | 9 | 2 | 1 | 1 | 1 | 1 | 1 | 1 | 1 | 2 | 1 | 2 | 1 | 2 | 1 |
| 15 | 10 | 1 | 1 | 3 | 3 | 3 | 3 | 3 | 3 | 4 | 4 | 4 | 4 | 4 | 4 |
| 16 | 10 | 2 | 1 | 3 | 3 | 3 | 3 | 3 | 3 | 4 | 4 | 4 | 4 | 4 | 4 |
| 17 | 11 | 1 | 1 | 2 | 2 | 2 | 2 | 2 | 3 | 3 | 3 | 3 | 3 | 3 | 3 |
| 18 | 11 | 2 | 1 | 2 | 2 | 2 | 2 | 2 | 2 | 3 | 3 | 3 | 3 | 3 | 3 |
| 19 | 12 | 1 | 1 | 2 | 2 | 2 | 2 | 2 | 2 | 2 | 2 | 2 | 2 | 2 | 2 |
| 20 | 13 | 1 | 1 | 2 | 2 | 2 | 2 | 2 | 2 | 3 | 3 | 3 | 3 | 3 | 3 |
| 21 | 13 | 2 | 1 | 3 | 3 | 3 | 3 | 3 | 3 | 4 | 4 | 4 | 4 | 4 | 4 |
| 22 | 14 | 1 | 1 | 2 | 2 | 2 | 2 | 1 | 2 | 2 | 2 | 2 | 2 | 2 | 2 |
| 23 | 14 | 2 | 1 | 2 | 2 | 1 | 1 | 2 | 2 | 2 | 2 | 2 | 2 | 2 | 2 |
| 24 | 15 | 2 | 1 | 2 | 2 | 2 | 2 | 2 | 2 | 3 | 3 | 3 | 3 | 3 | 3 |
| 25 | 16 | 1 | 1 | 2 | 2 | 2 | 2 | 2 | 2 | 2 | 2 | 2 | 2 | 2 | 2 |
| 26 | 17 | 1 | 1 | 2 | 2 | 2 | 2 | 3 | 3 | 3 | 3 | 3 | 3 | 3 | 3 |
| 27 | 17 | 2 | 1 | 3 | 3 | 3 | 3 | 3 | 3 | 3 | 3 | 3 | 3 | 3 | 3 |
| 28 | 18 | 1 | 1 | 2 | 2 | 2 | 2 | 2 | 2 | 3 | 3 | 3 | 3 | 3 | 3 |
| 29 | 19 | 1 | 1 | 1 | 1 | 1 | 1 | 1 | 1 | 2 | 2 | 2 | 2 | 2 | 2 |
| 30 | 19 | 2 | 1 | 1 | 1 | 1 | 1 | 2 | 2 | 2 | 2 | 2 | 2 | 2 | 2 |
| 31 | 20 | 1 | 1 | 2 | 2 | 2 | 2 | 2 | 2 | 2 | 2 | 2 | 2 | 2 | 2 |
| 32 | 20 | 2 | 1 | 2 | 2 | 2 | 2 | 2 | 2 | 2 | 2 | 2 | 2 | 2 | 2 |
| 33 | 21 | 2 | 1 | 2 | 2 | 2 | 2 | 2 | 2 | 2 | 2 | 2 | 2 | 3 | 3 |
| 34 | 22 | 1 | 1 | 1 | 1 | 1 | 1 | 1 | 2 | 2 | 2 | 2 | 2 | 2 | 2 |
| 35 | 22 | 2 | 1 | 1 | 1 | 1 | 1 | 1 | 1 | 2 | 2 | 2 | 2 | 2 | 2 |
| 36 | 23 | 1 | 1 | 2 | 2 | 2 | 2 | 3 | 3 | 3 | 3 | 3 | 3 | 3 | 3 |
| 37 | 23 | 2 | 1 | 2 | 2 | 2 | 2 | 2 | 2 | 3 | 3 | 2 | 3 | 3 | 3 |
| 38 | 24 | 2 | 1 | 2 | 2 | 2 | 2 | 2 | 2 | 2 | 2 | 3 | 3 | 3 | 3 |
| 39 | 25 | 1 | 1 | 3 | 3 | 3 | 3 | 3 | 3 | 3 | 3 | 3 | 3 | 3 | 3 |
| 40 | 25 | 2 | 1 | 3 | 3 | 3 | 3 | 3 | 3 | 3 | 3 | 3 | 3 | 3 | 3 |
| 41 | 26 | 1 | 1 | 1 | 1 | 1 | 1 | 1 | 1 | 2 | 2 | 2 | 2 | 2 | 2 |
| 42 | 26 | 2 | 1 | 1 | 1 | 1 | 1 | 2 | 2 | 2 | 2 | 2 | 2 | 2 | 2 |
| 43 | 27 | 1 | 1 | 2 | 2 | 2 | 2 | 3 | 2 | 3 | 3 | 3 | 3 | 2 | 3 |
| 44 | 28 | 2 | 1 | 4 | 4 | 4 | 4 | 4 | 4 | 4 | 4 | 4 | 4 | 4 | 4 |
| 45 | 29 | 1 | 1 | 2 | 2 | 2 | 2 | 2 | 2 | 2 | 2 | 2 | 2 | 2 | 2 |
| 46 | 29 | 2 | 1 | 2 | 2 | 2 | 2 | 2 | 2 | 2 | 2 | 2 | 2 | 2 | 2 |
| 47 | 30 | 1 | 1 | 2 | 2 | 2 | 2 | 2 | 2 | 3 | 3 | 3 | 3 | 3 | 3 |
| 48 | 30 | 2 | 1 | 2 | 2 | 2 | 2 | 2 | 2 | 2 | 2 | 3 | 3 | 3 | 3 |
| 49 | 31 | 1 | 1 | 3 | 3 | 3 | 3 | 3 | 3 | 4 | 4 | 4 | 4 | 4 | 4 |
| 50 | 31 | 2 | 1 | 3 | 3 | 3 | 3 | 3 | 3 | 4 | 4 | 4 | 4 | 4 | 4 |
| 51 | 32 | 2 | 1 | 2 | 2 | 2 | 2 | 2 | 2 | 3 | 3 | 3 | 3 | 3 | 3 |
| 52 | 33 | 1 | 1 | 2 | 2 | 2 | 2 | 2 | 2 | 3 | 3 | 3 | 3 | 3 | 3 |
| 53 | 33 | 2 | 1 | 3 | 2 | 2 | 2 | 2 | 2 | 3 | 3 | 3 | 3 | 3 | 3 |
| 54 | 34 | 1 | 1 | 2 | 2 | 2 | 2 | 2 | 2 | 3 | 3 | 3 | 3 | 3 | 3 |
| 55 | 34 | 2 | 1 | 2 | 2 | 2 | 2 | 2 | 2 | 3 | 3 | 3 | 3 | 3 | 3 |
| 56 | 35 | 1 | 1 | 3 | 3 | 3 | 3 | 3 | 3 | 3 | 3 | 3 | 3 | 3 | 3 |
| 57 | 36 | 1 | 1 | 2 | 2 | 2 | 2 | 2 | 2 | 2 | 2 | 2 | 2 | 2 | 3 |
| 58 | 37 | 1 | 1 | 1 | 1 | 1 | 1 | 1 | 1 | 2 | 2 | 2 | 2 | 2 | 2 |
| 59 | 37 | 2 | 1 | 1 | 1 | 1 | 1 | 1 | 1 | 2 | 2 | 2 | 2 | 2 | 2 |
| 60 | 38 | 1 | 1 | 3 | 3 | 3 | 3 | 3 | 3 | 4 | 4 | 4 | 4 | 4 | 4 |
| 61 | 39 | 1 | 1 | 2 | 2 | 2 | 2 | 2 | 2 | 3 | 3 | 3 | 3 | 3 | 3 |
| 62 | 39 | 2 | 1 | 2 | 2 | 2 | 2 | 2 | 2 | 3 | 3 | 3 | 3 | 3 | 3 |
| 63 | 40 | 2 | 1 | 3 | 3 | 3 | 3 | 2 | 2 | 3 | 3 | 3 | 3 | 3 | 3 |
| 64 | 41 | 1 | 1 | 2 | 2 | 2 | 2 | 2 | 2 | 3 | 3 | 3 | 3 | 3 | 3 |
| 65 | 42 | 1 | 1 | 2 | 2 | 2 | 2 | 2 | 2 | 3 | 3 | 3 | 3 | 3 | 3 |
| 66 | 42 | 2 | 1 | 3 | 3 | 3 | 3 | 3 | 3 | 4 | 4 | 4 | 4 | 4 | 4 |
| 67 | 43 | 1 | 1 | 2 | 2 | 2 | 2 | 2 | 2 | 3 | 3 | 3 | 3 | 3 | 3 |
| 68 | 43 | 2 | 1 | 1 | 1 | 1 | 1 | 2 | 1 | 2 | 2 | 2 | 2 | 2 | 2 |
| 69 | 44 | 1 | 1 | 1 | 1 | 1 | 1 | 1 | 1 | 2 | 2 | 2 | 2 | 2 | 2 |
| 70 | 45 | 1 | 1 | 2 | 2 | 2 | 2 | 2 | 2 | 2 | 2 | 2 | 2 | 2 | 2 |
| 71 | 45 | 2 | 1 | 2 | 2 | 2 | 2 | 2 | 2 | 3 | 3 | 3 | 3 | 3 | 3 |
| 72 | 46 | 1 | 1 | 3 | 3 | 3 | 3 | 2 | 2 | 3 | 3 | 3 | 3 | 3 | 3 |
| 73 | 46 | 2 | 1 | 3 | 3 | 3 | 3 | 3 | 3 | 3 | 3 | 3 | 3 | 3 | 3 |
| 74 | 47 | 1 | 1 | 2 | 2 | 2 | 2 | 2 | 2 | 3 | 3 | 3 | 3 | 3 | 3 |
| 75 | 47 | 2 | 1 | 3 | 3 | 3 | 3 | 3 | 3 | 4 | 4 | 4 | 4 | 4 | 3 |
| 76 | 48 | 2 | 1 | 2 | 2 | 2 | 2 | 2 | 2 | 3 | 3 | 3 | 3 | 3 | 3 |
| 77 | 49 | 2 | 1 | 3 | 3 | 3 | 3 | 3 | 3 | 3 | 3 | 3 | 3 | 3 | 3 |
| 78 | 50 | 1 | 1 | 2 | 2 | 2 | 2 | 2 | 2 | 2 | 2 | 2 | 2 | 2 | 2 |
| 79 | 51 | 2 | 1 | 3 | 3 | 3 | 3 | 3 | 3 | 4 | 3 | 3 | 3 | 3 | 3 |
| 80 | 52 | 2 | 1 | 2 | 2 | 2 | 2 | 2 | 2 | 3 | 3 | 3 | 3 | 3 | 3 |
| 81 | 53 | 1 | 1 | 3 | 3 | 3 | 3 | 3 | 3 | 3 | 3 | 3 | 3 | 3 | 3 |
| 82 | 53 | 2 | 1 | 2 | 2 | 2 | 2 | 2 | 2 | 3 | 3 | 3 | 3 | 3 | 3 |
| 83 | 54 | 1 | 1 | 2 | 2 | 2 | 2 | 2 | 2 | 2 | 2 | 2 | 2 | 2 | 2 |
| 84 | 55 | 1 | 1 | 2 | 2 | 2 | 2 | 2 | 2 | 3 | 3 | 3 | 3 | 3 | 3 |
| 85 | 56 | 2 | 1 | 4 | 4 | 4 | 4 | 4 | 4 | 4 | 4 | 4 | 4 | 4 | 4 |
| 86 | 57 | 1 | 1 | 3 | 3 | 3 | 3 | 2 | 2 | 3 | 3 | 3 | 3 | 3 | 3 |
| 87 | 58 | 2 | 1 | 1 | 1 | 1 | 1 | 1 | 1 | 2 | 2 | 2 | 2 | 2 | 2 |
| 88 | 59 | 1 | 1 | 1 | 1 | 1 | 1 | 1 | 1 | 2 | 2 | 2 | 2 | 2 | 2 |
| 89 | 59 | 2 | 1 | 1 | 1 | 1 | 1 | 1 | 1 | 2 | 2 | 2 | 2 | 2 | 2 |
| 90 | 60 | 1 | 1 | 3 | 3 | 3 | 3 | 2 | 3 | 3 | 3 | 3 | 3 | 3 | 3 |
| 91 | 60 | 2 | 1 | 2 | 2 | 2 | 2 | 2 | 2 | 3 | 3 | 3 | 3 | 3 | 3 |
| 92 | 61 | 1 | 1 | 4 | 4 | 4 | 4 | 4 | 4 | 4 | 4 | 4 | 4 | 4 | 4 |
| 93 | 61 | 2 | 1 | 3 | 3 | 3 | 3 | 4 | 4 | 4 | 4 | 4 | 4 | 4 | 4 |
| 94 | 62 | 2 | 1 | 2 | 2 | 2 | 2 | 2 | 2 | 3 | 3 | 3 | 3 | 3 | 3 |
| 95 | 63 | 1 | 1 | 3 | 3 | 3 | 3 | 3 | 3 | 4 | 4 | 4 | 4 | 4 | 4 |
| 96 | 64 | 1 | 1 | 1 | 1 | 1 | 1 | 1 | 1 | 2 | 2 | 2 | 2 | 2 | 2 |
| 97 | 65 | 1 | 1 | 1 | 2 | 2 | 2 | 1 | 1 | 2 | 2 | 2 | 2 | 2 | 2 |
| 98 | 65 | 2 | 1 | 1 | 1 | 1 | 1 | 1 | 1 | 2 | 2 | 2 | 2 | 2 | 2 |
| 99 | 66 | 2 | 1 | 2 | 2 | 2 | 2 | 2 | 2 | 3 | 3 | 3 | 3 | 3 | 2 |
| 100 | 67 | 1 | 1 | 2 | 2 | 2 | 2 | 2 | 2 | 3 | 3 | 3 | 3 | 3 | 3 |
| 101 | 67 | 2 | 1 | 2 | 2 | 2 | 2 | 2 | 2 | 3 | 3 | 3 | 3 | 3 | 3 |
| 102 | 68 | 2 | 1 | 1 | 1 | 2 | 2 | 1 | 1 | 2 | 2 | 2 | 2 | 2 | 2 |
| 103 | 69 | 1 | 1 | 2 | 2 | 2 | 2 | 2 | 2 | 3 | 3 | 3 | 3 | 3 | 3 |
| 104 | 70 | 1 | 1 | 2 | 2 | 2 | 2 | 2 | 2 | 3 | 3 | 3 | 3 | 3 | 3 |
| 105 | 70 | 2 | 1 | 1 | 1 | 1 | 1 | 1 | 1 | 2 | 2 | 2 | 2 | 2 | 2 |
| 106 | 71 | 1 | 1 | 2 | 2 | 2 | 2 | 2 | 2 | 3 | 3 | 3 | 3 | 3 | 3 |
| 107 | 71 | 2 | 1 | 3 | 3 | 3 | 3 | 3 | 3 | 3 | 3 | 3 | 3 | 3 | 3 |
| 108 | 72 | 1 | 1 | 3 | 3 | 3 | 3 | 3 | 3 | 3 | 3 | 3 | 3 | 3 | 3 |
| 109 | 72 | 2 | 1 | 2 | 2 | 2 | 2 | 2 | 2 | 2 | 2 | 2 | 2 | 2 | 2 |
| 110 | 73 | 2 | 1 | 2 | 2 | 2 | 2 | 2 | 2 | 2 | 2 | 2 | 2 | 2 | 2 |
| 111 | 74 | 1 | 1 | 1 | 1 | 1 | 1 | 1 | 1 | 2 | 2 | 2 | 2 | 2 | 2 |
| 112 | 75 | 1 | 1 | 3 | 2 | 2 | 2 | 2 | 2 | 2 | 2 | 2 | 2 | 2 | 2 |
| 113 | 75 | 2 | 1 | 2 | 2 | 2 | 2 | 2 | 2 | 3 | 3 | 3 | 3 | 3 | 3 |
| 114 | 76 | 2 | 1 | 3 | 3 | 3 | 3 | 3 | 3 | 3 | 3 | 3 | 3 | 3 | 3 |
| 115 | 77 | 1 | 1 | 2 | 2 | 2 | 2 | 2 | 2 | 2 | 2 | 2 | 2 | 2 | 3 |
| 116 | 77 | 2 | 1 | 3 | 3 | 3 | 3 | 3 | 3 | 3 | 3 | 3 | 3 | 3 | 3 |
| 117 | 78 | 1 | 1 | 3 | 3 | 3 | 3 | 3 | 3 | 4 | 4 | 4 | 4 | 4 | 4 |
| 118 | 78 | 2 | 1 | 2 | 2 | 2 | 2 | 2 | 2 | 3 | 3 | 3 | 3 | 3 | 3 |
| 119 | 79 | 1 | 1 | 2 | 2 | 2 | 2 | 2 | 2 | 3 | 3 | 2 | 2 | 3 | 3 |
| 120 | 79 | 2 | 1 | 1 | 1 | 1 | 1 | 2 | 2 | 2 | 2 | 2 | 2 | 2 | 2 |
| 121 | 80 | 2 | 1 | 2 | 2 | 2 | 2 | 2 | 2 | 2 | 2 | 2 | 2 | 2 | 2 |
| 122 | 81 | 1 | 1 | 3 | 3 | 3 | 3 | 3 | 3 | 4 | 4 | 4 | 4 | 4 | 4 |
| 123 | 81 | 2 | 1 | 1 | 1 | 1 | 1 | 1 | 1 | 2 | 2 | 2 | 2 | 2 | 2 |
| 124 | 82 | 2 | 1 | 2 | 2 | 2 | 2 | 2 | 2 | 2 | 2 | 2 | 2 | 2 | 2 |
| 125 | 83 | 1 | 1 | 2 | 2 | 2 | 2 | 2 | 2 | 3 | 3 | 3 | 3 | 3 | 2 |
| 126 | 84 | 1 | 1 | 2 | 2 | 2 | 2 | 2 | 2 | 3 | 3 | 3 | 3 | 3 | 3 |
| 127 | 84 | 2 | 1 | 2 | 2 | 2 | 2 | 2 | 2 | 3 | 3 | 3 | 3 | 3 | 3 |
| 128 | 85 | 1 | 1 | 3 | 3 | 3 | 3 | 3 | 3 | 3 | 3 | 3 | 3 | 3 | 3 |
| 129 | 86 | 1 | 1 | 1 | 1 | 1 | 1 | 1 | 1 | 2 | 2 | 2 | 2 | 2 | 2 |
| 130 | 86 | 2 | 1 | 1 | 1 | 1 | 1 | 1 | 1 | 2 | 2 | 2 | 2 | 2 | 2 |
| 131 | 87 | 1 | 1 | 1 | 1 | 2 | 2 | 1 | 1 | 2 | 2 | 2 | 2 | 2 | 2 |
| 132 | 87 | 2 | 1 | 2 | 2 | 2 | 2 | 2 | 2 | 2 | 3 | 3 | 3 | 3 | 3 |
| 133 | 88 | 2 | 1 | 2 | 2 | 2 | 2 | 2 | 2 | 3 | 3 | 3 | 3 | 3 | 3 |
| 134 | 89 | 1 | 1 | 2 | 2 | 2 | 2 | 2 | 2 | 3 | 3 | 3 | 3 | 3 | 3 |
| 135 | 89 | 2 | 1 | 2 | 2 | 2 | 2 | 2 | 2 | 3 | 3 | 3 | 3 | 3 | 3 |
| 136 | 90 | 1 | 1 | 1 | 1 | 1 | 1 | 1 | 1 | 2 | 2 | 2 | 2 | 2 | 2 |
| 137 | 90 | 2 | 1 | 3 | 3 | 3 | 3 | 3 | 3 | 3 | 3 | 3 | 3 | 3 | 3 |
| 138 | 91 | 1 | 1 | 2 | 2 | 2 | 2 | 2 | 2 | 2 | 2 | 3 | 3 | 2 | 2 |
| 139 | 92 | 1 | 1 | 2 | 2 | 2 | 2 | 2 | 2 | 3 | 3 | 3 | 3 | 3 | 3 |
| 140 | 92 | 2 | 1 | 2 | 2 | 2 | 2 | 2 | 2 | 3 | 3 | 3 | 3 | 3 | 3 |
| 141 | 93 | 2 | 1 | 3 | 3 | 3 | 3 | 3 | 3 | 4 | 4 | 4 | 4 | 4 | 4 |
| 142 | 94 | 1 | 1 | 1 | 1 | 1 | 1 | 1 | 1 | 2 | 2 | 2 | 2 | 2 | 2 |
| 143 | 94 | 2 | 1 | 2 | 2 | 2 | 2 | 2 | 2 | 2 | 2 | 2 | 2 | 2 | 2 |
| 144 | 95 | 2 | 1 | 2 | 2 | 2 | 2 | 2 | 2 | 3 | 3 | 3 | 3 | 3 | 3 |
| 145 | 96 | 1 | 1 | 3 | 3 | 3 | 3 | 3 | 3 | 3 | 3 | 3 | 3 | 3 | 4 |
| 146 | 97 | 1 | 1 | 2 | 2 | 2 | 2 | 2 | 2 | 3 | 3 | 3 | 3 | 3 | 3 |
| 147 | 97 | 2 | 1 | 1 | 1 | 1 | 1 | 1 | 1 | 2 | 2 | 2 | 2 | 2 | 2 |
| 148 | 98 | 1 | 1 | 1 | 1 | 1 | 1 | 1 | 1 | 2 | 2 | 2 | 2 | 2 | 2 |
| 149 | 98 | 2 | 1 | 2 | 2 | 2 | 2 | 2 | 2 | 3 | 3 | 3 | 3 | 3 | 3 |
| 150 | 99 | 1 | 1 | 1 | 1 | 1 | 1 | 1 | 1 | 2 | 2 | 2 | 2 | 2 | 2 |
| 151 | 99 | 2 | 1 | 1 | 1 | 1 | 1 | 1 | 1 | 2 | 2 | 2 | 2 | 2 | 2 |
| 152 | 100 | 1 | 1 | 2 | 2 | 2 | 2 | 3 | 2 | 3 | 3 | 3 | 3 | 3 | 3 |
| 153 | 100 | 2 | 1 | 2 | 2 | 2 | 2 | 2 | 2 | 2 | 2 | 2 | 2 | 2 | 2 |
| 154 | 101 | 1 | 1 | 4 | 4 | 4 | 4 | 4 | 4 | 4 | 4 | 4 | 4 | 4 | 4 |
| 155 | 101 | 2 | 1 | 4 | 4 | 4 | 4 | 4 | 4 | 4 | 4 | 4 | 4 | 4 | 4 |
| 156 | 102 | 2 | 1 | 3 | 3 | 3 | 3 | 3 | 3 | 3 | 3 | 3 | 3 | 3 | 3 |
| 157 | 103 | 1 | 1 | 3 | 3 | 3 | 3 | 3 | 3 | 4 | 4 | 4 |  | 4 | 4 |
| 158 | 104 | 1 | 1 | 3 | 3 | 3 | 3 | 4 | 4 | 4 | 4 | 4 | 4 | 4 | 4 |
| 159 | 105 | 1 | 1 | 3 | 3 | 3 | 3 | 3 | 3 | 3 | 3 | 3 | 3 | 3 | 3 |
| 160 | 105 | 2 | 1 | 3 | 3 | 3 | 3 | 3 | 3 | 3 | 3 | 3 | 3 | 3 | 4 |
| 161 | 106 | 1 | 1 | 4 | 4 | 4 | 4 | 4 | 4 | 4 | 4 | 4 | 4 | 4 | 4 |
| 162 | 107 | 1 | 1 | 4 | 4 | 4 | 4 | 4 | 4 | 4 | 4 | 4 | 4 | 4 | 4 |
| 163 | 107 | 2 | 1 | 3 | 3 | 3 | 3 | 4 | 4 | 3 | 4 | 3 | 4 | 3 | 4 |
| 164 | 108 | 1 | 1 | 3 | 3 | 3 | 3 | 3 | 3 | 3 | 3 | 3 | 3 | 4 | 3 |
| 165 | 108 | 2 | 1 | 3 | 3 | 3 | 3 | 3 | 3 | 4 | 4 | 4 | 4 | 4 | 4 |
| 166 | 109 | 2 | 1 | 3 | 3 | 3 | 3 | 3 | 3 | 3 | 3 | 3 | 3 | 3 | 3 |
| 167 | 110 | 1 | 1 | 3 | 3 | 3 | 3 | 3 | 3 | 3 | 3 | 3 | 3 | 3 | 3 |
| 168 | 110 | 2 | 1 | 4 | 4 | 4 | 4 | 4 | 4 | 4 | 4 | 4 | 4 | 4 | 4 |
| 169 | 111 | 2 | 1 | 3 | 3 | 3 | 3 | 3 | 3 | 3 | 3 | 3 | 3 | 3 | 3 |
| 170 | 112 | 1 | 1 | 4 | 4 | 4 | 4 | 4 | 4 | 4 | 4 | 4 | 4 | 4 | 4 |
| 171 | 112 | 2 | 1 | 3 | 3 | 3 | 3 | 3 | 3 | 4 | 4 | 4 | 4 | 4 | 4 |
| 172 | 113 | 1 | 1 | 4 | 4 | 4 | 4 | 3 | 3 | 4 | 4 | 4 | 4 | 4 | 4 |
| 173 | 114 | 1 | 1 | 3 | 3 | 3 | 3 | 3 | 3 | 4 | 4 | 4 | 4 | 4 | 4 |
| 174 | 114 | 2 | 1 | 3 | 3 | 3 | 3 | 3 | 3 | 3 | 3 | 3 | 3 | 3 | 3 |
| 175 | 115 | 2 | 1 | 3 | 3 | 3 | 3 | 3 | 3 | 4 | 4 | 4 | 4 | 4 | 4 |
| 176 | 116 | 1 | 1 | 4 | 4 | 4 | 4 | 4 | 4 | 4 | 4 | 4 | 4 | 4 | 4 |
| 177 | 116 | 2 | 1 | 3 | 3 | 3 | 3 | 3 | 4 | 4 | 4 | 4 | 4 | 4 | 4 |
| 178 | 117 | 1 | 1 | 3 | 3 | 3 | 3 | 3 | 3 | 3 | 3 | 3 | 3 | 3 | 3 |
| 179 | 118 | 1 | 1 | 3 | 3 | 3 | 3 | 3 | 3 | 4 | 4 | 4 | 4 | 4 | 4 |
| 180 | 118 | 2 | 1 | 3 | 3 | 3 | 3 | 3 | 3 | 3 | 3 | 3 | 3 | 3 | 3 |
| 181 | 119 | 1 | 1 | 3 | 3 | 4 | 4 | 3 | 3 | 4 | 4 | 4 | 4 | 4 | 4 |
| 182 | 119 | 2 | 1 | 4 | 4 | 4 | 4 | 4 | 4 | 4 | 4 | 4 | 4 | 4 | 4 |
| 183 | 120 | 2 | 1 | 3 | 3 | 3 | 3 | 3 | 3 | 4 | 4 | 4 | 4 | 4 | 4 |
| 184 | 121 | 1 | 1 | 4 | 4 | 4 | 4 | 4 | 4 | 4 | 4 | 4 | 4 | 4 | 4 |
| 185 | 122 | 1 | 1 | 3 | 3 | 3 | 3 | 3 | 3 | 3 | 3 | 3 | 3 | 3 | 3 |
| 186 | 123 | 2 | 1 | 4 | 4 | 4 | 4 | 4 | 4 | 4 | 4 | 4 | 4 | 4 | 4 |
| 187 | 124 | 1 | 1 | 3 | 3 | 3 | 3 | 3 | 4 | 3 | 3 | 3 | 3 | 3 | 3 |
| 188 | 124 | 2 | 1 | 3 | 3 | 3 | 3 | 3 | 3 | 4 | 4 | 4 | 4 | 4 | 4 |
| 189 | 125 | 1 | 1 | 4 | 4 | 4 | 4 | 4 | 4 | 4 | 4 | 4 | 4 | 4 | 4 |
| 190 | 126 | 2 | 1 | 3 | 3 | 3 | 3 | 4 | 4 | 4 | 4 | 4 | 4 | 4 | 4 |
| 191 | 127 | 1 | 1 | 3 | 3 | 3 | 3 | 3 | 3 | 3 | 3 | 3 | 3 | 3 | 3 |
| 192 | 127 | 2 | 1 | 4 | 4 | 4 | 4 | 4 | 4 | 4 | 4 | 4 | 4 | 4 | 4 |
| 193 | 128 | 1 | 1 | 3 | 3 | 3 | 3 | 3 | 3 | 4 | 4 | 4 | 4 | 4 | 4 |
| 194 | 129 | 1 | 1 | 3 | 3 | 3 | 3 | 3 | 3 | 3 | 3 | 3 | 3 | 3 | 3 |
| 195 | 129 | 2 | 1 | 3 | 3 | 3 | 3 | 3 | 3 | 3 | 4 | 4 | 4 | 4 | 4 |
| 196 | 130 | 2 | 1 | 3 | 3 | 3 | 3 | 3 | 3 | 3 | 3 | 3 | 3 | 3 | 3 |
| 197 | 131 | 1 | 1 | 3 | 3 | 3 | 3 | 3 | 3 | 4 | 4 | 4 | 4 | 4 | 4 |
| 198 | 131 | 2 | 1 | 4 | 4 | 4 | 4 | 4 | 4 | 4 | 4 | 4 | 4 | 4 | 4 |
| 199 | 132 | 1 | 1 | 4 | 4 | 4 | 4 | 4 | 4 | 4 | 4 | 4 | 4 | 4 | 4 |
| 200 | 132 | 2 | 1 | 3 | 3 | 3 | 3 | 3 | 3 | 4 | 4 | 4 | 4 | 4 | 4 |
| 201 | 133 | 2 | 1 | 3 | 3 | 3 | 3 | 3 | 3 | 3 | 3 | 3 | 3 | 3 | 3 |
| 202 | 134 | 1 | 1 | 3 | 3 | 3 | 3 | 4 | 3 | 4 | 4 | 4 | 4 | 4 | 4 |
| 203 | 134 | 2 | 1 | 4 | 4 | 4 | 4 | 4 | 4 | 4 | 4 | 4 | 4 | 4 | 4 |
| 204 | 135 | 1 | 1 | 2 | 2 | 2 | 2 | 2 | 2 | 3 | 3 | 3 | 3 | 3 | 3 |
| 205 | 136 | 1 | 1 | 3 | 3 | 3 | 3 | 3 | 3 | 4 | 4 | 4 | 4 | 4 | 4 |
| 206 | 136 | 2 | 1 | 4 | 4 | 4 | 4 | 4 | 4 | 4 | 4 | 4 | 4 | 4 | 4 |
| 207 | 137 | 2 | 1 | 2 | 2 | 2 | 2 | 2 | 2 | 3 | 3 | 3 | 3 | 3 | 3 |
| 208 | 138 | 1 | 1 | 3 | 3 | 3 | 3 | 3 | 3 | 3 | 3 | 3 | 3 | 3 | 3 |
| 209 | 139 | 1 | 1 | 3 | 3 | 3 | 3 | 4 | 3 | 4 | 4 | 4 | 4 | 4 | 4 |
| 210 | 140 | 1 | 1 | 2 | 2 | 2 | 2 | 2 | 2 | 3 | 3 | 3 | 3 | 3 | 3 |
| 211 | 140 | 2 | 1 | 3 | 3 | 3 | 3 | 3 | 3 | 3 | 3 | 3 | 3 | 3 | 3 |
| 212 | 141 | 2 | 1 | 3 | 3 | 3 | 3 | 3 | 3 | 3 | 4 | 4 | 4 | 4 | 4 |
| 213 | 142 | 2 | 1 | 3 | 3 | 3 | 3 | 3 | 3 | 4 | 4 | 4 | 4 | 4 | 4 |
| 214 | 143 | 1 | 1 | 2 | 2 | 2 | 2 | 2 | 2 | 2 | 2 | 2 | 2 | 2 | 2 |
| 215 | 143 | 2 | 1 | 2 | 2 | 2 | 2 | 2 | 2 | 3 | 3 | 3 | 3 | 3 | 3 |
| 216 | 144 | 1 | 1 | 2 | 2 | 2 | 2 | 2 | 2 | 3 | 3 | 3 | 3 | 3 | 3 |
| 217 | 145 | 2 | 1 | 2 | 2 | 2 | 2 | 3 | 3 | 3 | 3 | 3 | 3 | 3 | 3 |
| 218 | 146 | 1 | 1 | 3 | 3 | 3 |  | 3 | 3 | 3 | 3 | 3 | 3 | 3 | 3 |
| 219 | 147 | 1 | 1 | 2 | 2 | 2 | 2 | 2 | 2 | 2 | 2 | 2 | 2 | 2 | 2 |
| 220 | 147 | 2 | 1 | 2 | 2 | 2 | 2 | 2 | 2 | 3 | 3 | 3 | 3 | 3 | 3 |
| 221 | 148 | 1 | 1 | 2 | 2 | 2 | 2 | 2 | 2 | 3 | 3 | 3 | 3 | 3 | 3 |
| 222 | 149 | 1 | 1 | 2 | 2 | 2 | 2 | 2 | 2 | 2 | 2 | 2 | 2 | 2 | 2 |
| 223 | 149 | 2 | 1 | 2 | 2 | 2 | 2 | 2 | 2 | 3 | 3 | 3 | 3 | 3 | 3 |
| 224 | 150 | 2 | 1 | 3 | 3 | 3 | 3 | 3 | 3 | 4 | 4 | 4 | 4 | 4 | 4 |
| 225 | 151 | 1 | 1 | 4 | 4 | 4 | 4 | 4 | 4 | 4 | 4 | 4 | 4 | 4 | 4 |
| 226 | 152 | 2 | 1 | 2 | 2 | 2 | 2 | 2 | 2 | 3 | 3 | 3 | 3 | 3 | 3 |
| 227 | 153 | 1 | 1 | 2 | 2 | 2 | 2 | 2 | 2 | 2 | 2 | 2 | 2 | 2 | 2 |
| 228 | 153 | 2 | 1 | 2 | 2 | 2 | 2 | 3 | 2 | 3 | 3 | 3 | 3 | 3 | 3 |
| 229 | 154 | 1 | 1 | 4 | 4 | 4 | 4 | 4 | 4 | 4 | 4 | 4 | 4 | 4 | 4 |
| 230 | 155 | 1 | 1 | 2 | 2 | 2 | 2 | 2 | 2 | 3 | 3 | 3 | 3 | 3 | 3 |
| 231 | 156 | 2 | 1 | 2 | 2 | 2 | 2 | 2 | 2 | 3 | 3 | 3 | 3 | 3 | 3 |
| 232 | 157 | 1 | 1 | 3 | 3 | 3 | 3 | 3 | 3 | 3 | 3 | 3 | 3 | 3 | 3 |
| 233 | 157 | 2 | 1 | 2 | 2 | 2 | 2 | 2 | 2 | 3 | 3 | 3 | 3 | 3 | 3 |
| 234 | 158 | 1 | 1 | 2 | 2 | 2 | 2 | 2 | 2 | 3 | 3 | 3 | 3 | 3 | 3 |
| 235 | 159 | 2 | 1 | 3 | 3 | 3 | 3 | 3 | 3 | 4 | 4 | 4 | 4 | 4 | 4 |
| 236 | 160 | 1 | 1 | 2 | 2 | 2 | 2 | 2 | 2 | 3 | 3 | 3 | 3 | 3 | 3 |
| 237 | 160 | 2 | 1 | 4 | 4 | 4 | 4 | 3 | 4 | 4 | 4 | 4 | 4 | 4 | 4 |
| 238 | 161 | 2 | 1 | 3 | 3 | 3 | 3 | 3 | 3 | 3 | 3 | 3 | 3 | 3 | 3 |
| 239 | 162 | 1 | 1 | 3 | 3 | 3 | 3 | 3 | 3 | 4 | 4 | 4 | 4 | 4 | 4 |
| 240 | 163 | 1 | 1 | 4 | 4 | 4 | 4 | 4 | 4 | 4 | 4 | 4 | 4 | 4 | 4 |
| 241 | 163 | 2 | 1 | 2 | 2 | 2 | 2 | 2 | 2 | 2 | 2 | 2 | 2 | 2 | 2 |
| 242 | 164 | 2 | 1 | 3 | 3 | 3 | 3 | 3 |  | 3 | 3 | 3 | 3 | 3 | 3 |
| 243 | 165 | 2 | 1 | 2 | 2 | 2 | 2 | 2 | 2 | 2 | 3 | 3 | 3 | 3 | 3 |
| 244 | 166 | 1 | 1 | 2 | 2 | 2 | 2 | 3 | 3 | 3 | 3 | 3 | 3 | 3 | 3 |
| 245 | 167 | 1 | 1 | 1 | 1 | 1 | 1 | 1 | 1 | 2 | 2 | 2 | 2 | 2 |  |
| 246 | 167 | 2 | 1 | 3 | 3 | 3 | 3 | 3 | 3 | 4 | 4 | 4 | 4 | 4 | 4 |
| 247 | 168 | 1 | 1 | 3 | 3 | 3 | 3 | 4 | 3 | 4 | 4 | 4 | 4 | 4 | 4 |
| 248 | 168 | 2 | 1 | 2 | 2 | 2 | 2 | 2 | 2 | 2 | 2 | 2 | 2 | 2 | 2 |
| 249 | 169 | 1 | 1 | 2 | 2 | 2 | 2 | 2 | 2 | 2 | 2 | 2 | 2 | 2 | 2 |
| 250 | 169 | 2 | 1 | 3 | 3 | 3 | 3 | 3 | 3 | 4 | 4 | 4 | 4 | 4 | 4 |
| 251 | 170 | 1 | 1 | 2 | 2 | 2 | 2 | 2 | 2 | 3 | 3 | 3 | 3 | 3 | 3 |
| 252 | 170 | 2 | 1 | 3 | 3 | 3 | 3 | 3 | 3 | 4 | 4 | 4 | 4 | 4 | 4 |
| 253 | 171 | 1 | 1 | 1 | 1 | 1 | 1 | 1 | 1 | 2 | 2 | 2 | 2 | 2 | 2 |
| 254 | 171 | 2 | 1 | 3 | 3 | 3 | 3 | 3 | 3 | 4 | 4 | 4 | 4 | 4 | 4 |
| 255 | 172 | 1 | 1 | 4 | 4 | 4 | 4 | 4 | 4 | 4 | 4 | 4 | 4 | 4 | 4 |
| 256 | 172 | 2 | 1 | 1 | 1 | 2 | 2 | 2 | 2 | 2 | 2 | 2 | 2 | 2 | 2 |
| 257 | 173 | 1 | 2 | 1 | 1 | 1 | 2 | 2 | 2 | 2 | 2 | 2 | 2 | 2 | 2 |
| 258 | 173 | 2 | 2 | 1 | 1 | 1 | 1 | 1 | 2 | 2 | 2 | 2 | 2 | 2 | 2 |
| 259 | 174 | 1 | 2 | 1 | 1 | 2 | 1 | 1 | 1 | 2 | 2 | 2 | 2 | 2 | 2 |
| 260 | 174 | 2 | 2 | 1 | 1 | 1 | 1 | 1 | 1 | 2 | 2 | 2 | 2 | 2 | 2 |
| 261 | 175 | 2 | 2 | 2 | 2 | 2 | 2 | 2 | 2 | 2 | 2 | 2 | 2 | 2 | 3 |
| 262 | 176 | 1 | 2 | 2 | 2 | 2 | 2 | 2 | 1 | 2 | 2 | 2 | 2 | 2 | 2 |
| 263 | 176 | 2 | 2 | 2 | 2 | 2 | 2 | 2 | 2 | 3 | 3 | 3 | 3 | 3 | 3 |
| 264 | 177 | 1 | 2 | 2 | 2 | 2 | 2 | 2 | 2 | 3 | 3 | 3 | 3 | 3 | 3 |
| 265 | 178 | 1 | 2 | 3 | 3 | 3 | 3 | 4 | 3 | 4 | 4 | 4 | 4 | 4 | 4 |
| 266 | 179 | 2 | 2 | 2 | 2 | 2 | 2 | 2 | 2 | 3 | 3 | 3 | 3 | 3 | 3 |
| 267 | 180 | 1 | 2 | 3 | 3 | 4 | 4 | 3 | 3 | 4 | 4 | 4 | 4 | 4 | 4 |
| 268 | 180 | 2 | 2 | 4 | 4 | 4 | 4 | 4 | 4 | 4 | 4 | 4 | 4 | 4 | 4 |
| 269 | 181 | 1 | 2 | 4 | 4 | 4 | 4 | 4 | 4 | 3 | 3 | 3 | 3 | 3 | 3 |
| 270 | 181 | 2 | 2 | 2 | 2 | 2 | 2 | 2 | 2 | 2 | 2 | 3 | 3 | 3 | 3 |
| 271 | 182 | 1 | 2 | 3 | 3 | 3 | 3 | 3 | 3 | 4 | 4 | 4 | 4 | 4 | 4 |
| 272 | 182 | 2 | 2 | 1 | 1 | 1 | 1 | 1 | 1 | 2 | 2 | 2 | 2 | 2 | 2 |
| 273 | 183 | 2 | 2 | 2 | 2 | 2 | 2 | 2 | 2 | 3 | 3 | 3 | 3 | 3 | 3 |
| 274 | 184 | 1 | 2 | 1 | 1 | 1 | 1 | 1 | 1 | 2 | 2 | 2 | 2 | 2 | 2 |
| 275 | 185 | 1 | 2 | 2 | 2 | 2 | 2 | 2 | 2 | 3 | 2 | 3 | 3 | 3 | 3 |
| 276 | 185 | 2 | 2 | 3 | 3 | 3 | 3 | 3 | 3 | 3 | 3 | 3 | 3 | 3 | 3 |
| 277 | 186 | 2 | 2 | 2 | 2 | 2 | 2 | 2 | 3 | 3 | 3 | 3 | 3 | 3 | 3 |
| 278 | 187 | 1 | 2 | 3 | 3 | 2 | 3 | 3 | 3 | 3 | 3 | 3 | 3 | 3 | 3 |
| 279 | 188 | 1 | 2 | 3 | 3 | 3 | 3 | 3 | 3 | 3 | 3 | 3 | 3 | 3 | 3 |
| 280 | 188 | 2 | 2 | 3 | 3 | 3 | 3 | 3 | 3 | 3 | 3 | 3 | 3 | 3 | 3 |
| 281 | 189 | 2 | 2 | 2 | 2 | 2 | 2 | 2 | 2 | 3 | 3 | 3 | 3 | 3 | 3 |
| 282 | 190 | 1 | 2 | 4 | 4 | 4 | 4 | 4 | 4 | 4 | 4 | 4 | 4 | 4 | 4 |
| 283 | 190 | 2 | 2 | 2 | 2 | 2 | 2 | 2 | 2 | 3 | 3 | 3 | 3 | 3 | 3 |
| 284 | 191 | 1 | 2 | 2 | 2 | 2 | 2 | 2 | 2 | 2 | 2 | 2 | 2 | 2 | 2 |
| 285 | 192 | 1 | 2 | 2 | 2 | 2 |  | 3 | 2 | 3 | 3 | 3 | 3 | 3 | 3 |
| 286 | 192 | 2 | 2 | 2 | 2 | 2 | 2 | 2 | 2 | 3 | 3 | 3 | 3 | 3 | 3 |
| 287 | 193 | 2 | 2 | 1 | 1 | 1 | 1 | 1 | 1 | 2 | 2 | 2 | 2 | 2 | 2 |
| 288 | 194 | 1 | 2 | 1 | 1 | 1 | 1 | 1 | 1 | 2 | 2 | 2 | 2 | 2 | 2 |
| 289 | 194 | 2 | 2 | 2 | 2 | 2 | 2 | 2 | 2 | 2 | 3 | 3 | 3 | 3 | 3 |
| 290 | 195 | 1 | 2 | 3 | 3 | 3 | 3 | 3 | 3 | 3 | 3 | 3 | 3 | 3 | 3 |
| 291 | 195 | 2 | 2 | 3 | 3 | 3 | 3 | 3 | 3 | 4 | 4 | 4 | 4 | 4 | 4 |
| 292 | 196 | 1 | 2 | 2 | 2 | 2 | 2 | 2 | 2 | 3 | 3 | 3 | 3 | 3 | 3 |
| 293 | 196 | 2 | 2 | 1 | 1 | 1 | 1 | 1 | 1 | 2 | 2 | 2 | 2 | 2 | 2 |
| 294 | 197 | 1 | 2 | 3 | 3 | 3 | 3 | 3 | 3 | 4 | 4 | 4 | 4 | 4 | 4 |
| 295 | 198 | 2 | 2 | 2 | 2 | 2 | 2 | 2 | 2 | 2 | 2 | 2 | 2 | 2 | 2 |
| 296 | 199 | 2 | 2 | 2 | 2 | 2 | 2 | 2 | 2 | 3 | 3 | 3 | 3 | 3 | 4 |
| 297 | 200 | 1 | 2 | 2 | 2 | 2 | 2 | 3 | 2 | 3 | 3 | 3 | 3 | 3 | 3 |
| 298 | 201 | 1 | 2 | 3 | 3 | 3 | 3 | 3 | 3 | 4 | 3 | 4 | 3 | 4 | 3 |
| 299 | 201 | 2 | 2 | 1 | 1 | 1 | 1 | 1 | 1 | 2 | 2 | 2 | 2 | 2 | 2 |
| 300 | 202 | 1 | 2 | 2 | 2 | 2 | 2 | 2 | 2 | 3 | 3 | 3 | 3 | 3 | 3 |
| 301 | 202 | 2 | 2 | 3 | 3 | 3 | 3 | 4 | 3 | 4 | 4 | 4 | 4 | 4 | 4 |
| 302 | 203 | 1 | 2 | 2 | 2 | 2 | 2 | 2 | 2 | 2 | 2 | 2 | 2 | 2 | 2 |
| 303 | 203 | 2 | 2 | 2 | 2 | 2 | 2 | 2 | 2 | 3 | 3 | 3 | 3 | 3 | 3 |
| 304 | 204 | 2 | 2 | 3 | 3 | 3 | 3 | 3 | 3 | 4 | 4 | 4 | 4 | 4 | 4 |
| 305 | 205 | 1 | 2 | 3 | 3 | 3 | 3 | 4 | 3 | 4 | 4 | 4 | 4 | 4 | 4 |
| 306 | 206 | 1 | 2 | 3 | 3 | 3 | 3 | 3 | 3 | 3 | 3 | 3 | 3 | 3 | 3 |
| 307 | 206 | 2 | 2 | 2 | 2 | 2 | 2 | 2 | 2 | 2 | 2 | 2 | 2 | 2 | 2 |
| 308 | 207 | 1 | 2 | 2 | 2 | 2 | 2 | 2 | 2 | 2 | 2 | 2 | 2 | 2 | 2 |
| 309 | 208 | 1 | 2 | 3 | 3 | 3 | 3 | 4 | 3 | 4 | 4 | 4 | 4 | 4 | 4 |
| 310 | 208 | 2 | 2 | 2 | 2 | 2 | 2 | 2 | 2 | 3 | 3 | 3 | 3 | 3 | 3 |
| 311 | 209 | 1 | 1 | 1 | 1 | 1 | 1 | 1 | 1 | 2 | 2 | 2 | 2 | 2 | 2 |
| 312 | 209 | 2 | 2 | 4 | 4 | 4 | 4 | 4 | 4 | 4 | 4 | 4 | 4 | 4 | 4 |
| 313 | 210 | 1 | 2 | 3 | 3 | 3 | 3 | 3 | 3 | 4 | 4 | 4 | 4 | 4 | 4 |
| 314 | 210 | 2 | 1 | 2 | 2 | 2 | 2 | 2 | 2 | 3 | 3 | 3 | 3 | 3 | 3 |
| 315 | 211 | 1 | 1 | 2 | 2 | 2 | 2 | 2 | 2 | 3 | 3 | 3 | 3 | 3 | 3 |
| 316 | 211 | 2 | 2 | 4 | 4 | 4 | 4 | 4 | 4 | 4 | 4 | 4 | 4 | 4 | 4 |
| 317 | 212 | 1 | 1 | 1 | 1 | 1 | 1 | 1 | 1 | 2 | 2 | 2 | 2 | 2 | 2 |
| 318 | 212 | 2 | 2 | 3 | 3 | 3 | 3 | 3 | 3 | 4 | 4 | 4 | 4 | 4 | 4 |
